# Secretome profiling of *Artemisia absinthium* extract-loaded polymeric nanoparticle-treated MCF-7 and MDA-MB-231 revealed perturbation in microtubule assembly and cell migration

**DOI:** 10.3389/fonc.2023.1209168

**Published:** 2023-08-31

**Authors:** Sana Kauser, Mohd Mughees, Irengbam Rocky Mangangcha, Sanskriti Swami, Saima Wajid

**Affiliations:** ^1^ Department of Biotechnology, School of Chemical and Life Sciences, Jamia Hamdard, New Delhi, India; ^2^ Department of Zoology, Deshbandhu College, University of Delhi, Delhi, India

**Keywords:** *Artemisia absinthium*, nano LC-MS/MS, GENT2, GSCA, GDSC, CTRP

## Abstract

**Introduction:**

*Artemisia absinthium* (wormwood) exhibits anticancer properties by inhibiting proliferation and causing cell death in breast cancer. Targeted drug delivery of *A. absinthium* nanoformulation using N-isopropyl acrylamide, N-vinyl pyrrolidone, and acrylic acid-based polymeric nanoparticles (NVA-AA NPs) was ensured by utilizing features of the tumor microenvironment, although their mechanism of action involved in cytotoxicity remains unknown.

**Methods:**

The present study employed nano LC-MS/MS to identify differences in secretory protein expression associated with the treatment of breast cancer cell lines (MCF-7; MDA-MB-231) by NVA-AA NPs for the determination of affected pathways and easily accessible therapeutic targets. Different bioinformatics tools were used to identify signature differentially expressed proteins (DEPs) using survival analysis by GENT2 and correlation analysis between their mRNA expressions and sensitivity toward small-molecule drugs as well as immune cell infiltration by GSCA.

**Results:**

Analysis by GENT2 revealed 22 signature DEPs with the most significant change in their expression regulation, namely, gelsolin, alpha-fetoprotein, complement component C3, C7, histone H2B type 1-K, histone H2A.Z, H2AX, heat shock cognate 71 kDa protein, heat shock 70 kDa protein 1-like, cytochrome c somatic, GTP-binding nuclear protein Ran, tubulin beta chain, tubulin alpha-1B chain, tubulin alpha-1C chain, phosphoglycerate mutase 1, kininogen 1, carboxypeptidase N catalytic chain, fibulin-1, peroxiredoxins 4, lactate dehydrogenase C, SPARC, and SPARC-like protein 1. Correlation analysis between their mRNA expressions versus immune cell infiltrates showed a positive correlation with antitumor immune response elicited by these NPs as well as a correlation with drug response shown by the GDSC and CTRP drugs in different cancer cells.

**Discussion:**

Our results suggest that NVA-AA NPs were able to invade the tumor microenvironment; transformed the communication network between the cancer cells; affected potential drivers of microtubular integrity, nucleosome assembly, and cell cycle; and eventually caused cell death.

## 1 Introduction

Breast cancer is one of the most common and leading causes of mortality among women worldwide. According to an estimation by the American Cancer Society, 1.9 million new cancer cases were diagnosed with 609,360 cancer deaths in the United States in 2022 (1). Several conventional therapeutic interventions are available for breast cancer that mainly include chemotherapy, radiotherapy, mastectomy, hormone therapy, immunotherapy, or a combination of any of these (2). These treatment modalities such as chemotherapy are often associated with acute side effects such as hair fall, nausea, vomiting, cytopenia, cardiotoxicity, ovarian failure, chances of tumor relapse, and drug resistance among patients. Radiotherapy has proven to be effective with low chances of recurrence and high overall survival; however, radiation dermatitis is reported in patients (3). Mastectomy impacts the psychological well-being of patients and their quality of life (4). Similarly, hormone therapy also poses challenges among patients that include hypertension, osteoporosis, thromboembolic events, endometrial cancer, and dyspareunia (5). Different immune therapeutic strategies of immunotherapy targeted against different breast cancer subtypes have been developed that mainly deal with the recruitment of targeted antibodies or immune checkpoint inhibitors (ICI) aimed at triggering or restoring the immune responses against tumor cells. Different clinical subtypes of breast cancer vary in their immunological characteristics, comprising of tumor-infiltrating lymphocytes (TILs CD4, CD8), expression of programmed death ligand-1 (PD-L1), leukemia inhibitory factor (LIF), tumor-associated antigen, and random gene mutations (6). The monoclonal antibodies target molecular cell surface receptors of tumor cells enabling blockade of proliferative pathways (e.g., HER2, PD-L1) or tumor-infiltrating immune cell subsets in the tumor microenvironment hampering the activation of immune cells by tumor cells and abolishing the tumor. Already existing antitumor immune response elicited by TILs in the tumor microenvironment was associated with good responsiveness toward chemotherapy and prognosis (7). Similarly, other immunomarkers (PD-L1 or LIF) are also predictive indicators of the clinical outcome of ICI therapy. A number of drugs are clinically approved for targeted therapies against HER2+, HR+/HER2−, TNBC, and gBRCAm that include trastuzumab, neratinib, everolimus, abemaciclib, pembrolizumab, olaparib, and talazoparib (8). However, different targeted drugs in combination with chemotherapy for varying breast cancer subtypes showed a limited success rate and significant discrepancies in immune-modulatory response, efficacy, safety, and prognosis (9).

Sonodynamic therapy (SDT) is a new therapeutic strategy employing ultrasound for the activation of sonosensitizers that produce reactive oxygen species which could destroy tumor cells; however, a hypoxic tumor microenvironment poses certain limitations to SDT. Nanoplatforms are a widely explored biocompatible delivery system for treating different types of cancer that could considerably increase the efficiency of existing treatment by utilizing the tumor microenvironment. Recently, ultrasmall titanium nitride nanodots (TiNs) and titanium dioxide (TiO_2_)-based hollow nanoshells have been developed which act as sonosensitizers and carriers of free radical generators or hypoxia-selective cytotoxin, synergistically increasing the therapeutic efficiency of SDT against solid tumors (10–12). Similarly, photothermal therapy (PPT) is another strategy that uses near-infrared waves to eliminate solid tumors. Ultrasmall zirconium carbide nanodots (NDs) and mesoporous NiS_2_ nanospheres (mNiS_2_ NSs) have been designed that significantly improved the PTT effect by inhibiting tumor growth *in vitro* and *in vivo* (13, 14).

It is estimated that approximately 75% of anticancer drugs are designed and prepared from plant-derived natural products (15). *Artemisia absinthium* L. (wormwood) is a perennial herb that belongs to the family Asteraceae and is found all over the world such as in the Indian subcontinent. This plant has been used in ethnic medicine due to its several medicinal properties such as antiseptic, antibacterial, antifungal, antiparasitic, antihelminthic, and anti-inflammatory. The anticancer effect of *A. absinthium* methanolic extract has been studied *in-vitro* on human breast carcinoma cell lines MDA-MB-231 and MCF-7, depicting its antiproliferative effect mediated *via* the apoptotic pathway (16). Previously, our research group evaluated the therapeutic potential of *A. absinthium* ethanolic extract-loaded polymeric nanoparticles (NVA-AA) on breast cancer cell lines (MCF-7 and MDA-MB-231) targeting the tumor microenvironment. Targeted drug delivery involves the simultaneous administration of anticancer agents or phytoconstituents with various routes of action in order to combat the aforementioned drawbacks of the majority of anticancer medications. Temperature- and pH-sensitive, self-assembled polymeric nanoparticles, made of N-isopropyl acrylamide (NIPAAM), N-vinyl pyrrolidone (VP), and acrylic acid (AA), are biocompatible and have been used in the past with several drugs (e.g., ketorolac, riluzole) for site-specific delivery of different formulations (17, 18). Free radical polymerization was used to prepare NIPAAM, VP, and AA-based polymeric nanoparticles (NVAs). In our previous study, the cytotoxic potential of *A. absinthium* extract-loaded polymeric nanoparticles (NVA-AAs) was explored against breast cancer with a proteomic analysis approach to find out their mechanism of action. These NPs were able to cause significant cell proliferation inhibition, cell death, and cell cycle arrest at the G0/G1 phase in MCF-7 as well as MDA-MB-231 cell lines (19).

Secretome is the group of proteins secreted in the extracellular space by a cell, tissue, organ, or organism within the local environment which comprises an extracellular matrix (ECM) and is mainly responsible for cell–cell communication. Altered patterns of secretion are closely associated with the growth and progression of cancer as these transformed signals aid the cancer cells to metastasize. Altered cancer secretome tends to have an important role in tumor microenvironment communication as well as the development of chemoresistance (20). Moreover, the secretome is considered a reservoir of potential cancer biomarkers as well as drug and therapeutic targets involved in the molecular cascade of drug development (21). Therefore, the investigation of proteins secreted in the medium by *in-vitro*-cultured cancer cells will provide insight into the interaction of NVA-AA with the tumor microenvironment, unraveling easily accessible biomarkers and potential therapeutic targets.

Secretome profiling of the cell lines through the proteomics approach helps in the identification and selection of secreted proteins into the extracellular milieu to be utilized as biomarkers or drug targets for therapeutic application. The nano liquid chromatography-tandem mass spectrometry (nano LC-MS/MS) approach has been used previously for secretome profiling of different cell lines that helps in the quantification of hundreds to thousands of secretory proteins present in the media (22). In the present study, the nano LC-MS/MS proteomic approach was employed to compare the secretome of NVA-AA-treated MCF-7 and MDA-MB-231 cell lines with the corresponding untreated cell lines as a control to identify potential therapeutic targets among differentially expressed secretory proteins. A total of 208 and 194 specific proteins were identified in the MCF-7 and MDA-MB-231 cell lines, among which 65 and 156 proteins were significantly differentially expressed in the MCF-7 and MDA-MB-231 cell lines, respectively. In MCF-7, some of the upregulated proteins are reported to be involved in apoptosis, vesicular trafficking, inhibition of angiogenesis, cell proliferation, and metastasis. Some tumor suppressor genes were also highly upregulated in NVA-AA-treated cells. At the same time, a few proteins involved in tumorigenesis were downregulated by NVA-AA treatment in MCF-7 cells. Similarly, proteins involved in vesicular trafficking and inhibition of cancer invasion were highly upregulated in NVA-AA NP-treated MDA-MB-231 cells, while proteins involved in proliferation, tumorigenesis, proteasomal degradation, cell adhesion, and migration were downregulated after NVA-AA NP treatment.

## 2 Materials and methods

### 2.1 Chemicals

N-isopropylacrylamide (NIPAAM) was procured from Sigma-Aldrich, USA and recrystallized with N-hexane overnight. N-vinyl 2-pyrrolidone (VP) and acrylic acid (AA) were purchased from Across Organics (USA). Dulbecco’s modified Eagle’s medium (DMEM), fetal bovine serum (FBS), antibiotic solution (100×), trypsin (with 0.5% EDTA), and ammonium persulfate (APS) were bought from HiMedia, USA, and ferrous ammonium sulfate (FAS) was from SRL Pvt Ltd. India MCF-7 and MDA-MB-231 cell lines were procured from the National Centre for Cell Science (NCCS), Pune, India.

### 2.2 Preparation of *Artemisia absinthium* ethanolic extract-loaded NIPAAM-VP-AA polymeric nanoparticles

NIPAAM-VP-AA (NVA-AA) polymeric nanoparticles were synthesized based on previous studies (19, 23, 24). The monomers N-isopropyl acrylamide (NIPAAM), N-vinylpyrrolidone (VP), and acrylic acid (AA) were employed in the molar ratio of 90:10:5 for free radical polymerization. N,N′-methylene-bisacrylamide (MBA) was used as a cross-linker, FAS as an activator, and APS as an initiator. One hundred eighty milligrams of NIPAAM, 20 µl of VP, and 10 µl of AA were dissolved in 20 ml of double distilled water accompanied by vigorous vortexing. One hundred microliters of MBA (0.049 g/ml), 60 µl of FAS (5 mg/ml), and 100 µl of APS (saturated) were added to trigger the polymerization reaction, and nitrogen gas was passed to maintain an inert environment for 24 h at 32°C. The final solution was dialyzed through a cellulose dialyzing membrane (cutoff 12 kDa) and characterized for the determination of the average size distribution and morphology of the synthesized NPs. The uniform, monodisperse, and spherical-shaped NPs were characterized by dynamic scattering light (DLS) spectroscopy and transmission electron microscopy (TEM)*. Artemisia absinthium* ethanolic extract was added slowly to the aqueous solution of NPs with continuous vortexing and mild sonication to assist its physical entrapment into the hydrophobic core of NPs.

### 2.3 Cell culture

The selection of relevant breast cancer cell lines for the translational study was done as per the a) molecular subtype, b) metastatic status, c) best or worst prognosis, and d) response rate to different therapies. Two subtypes were further chosen out of the four existing breast cancer subtypes: the first is luminal A subtype which is ER and/or PR+, HER2−, slow growing with a low-grade, high response rate, and the finest prognosis, and the second was triple-negative breast cancer (TNBC) subtype which is ER−, PR−, and HER2−, aggressive in nature with a low response rate and a variable prognosis. Therefore, MCF-7 and MDA-MB-231 were selected for the luminal A and the TNBC subtype, respectively. MCF-7 and MDA-MB-231 cells were grown in DMEM supplemented with 10% FBS at 37°C and 5% CO_2_. Cells (10,000 cells per well) were grown onto 96-well plates and treated with an IC_50_ concentration of *A. absinthium* ethanolic extract-loaded NIPAAM-VP-AA (NVA-AA) polymeric nanoparticles at 176.83 ± 11.8 μg/ml for MCF-7 and 181.39 ± 23.2 μg/ml for MDA-MB-231 (19). After 24 h, the conditioned media were collected and centrifuged for removing debris. The supernatants were concentrated by lyophilization and the protein was quantified by Bradford’s method.

### 2.4 Sample preparation

Samples were dissolved in 6 M of Gn-HCl (50 mM Tris, pH 8.8) buffer. A 50-µg sample was reduced with 5 mM of TCEP and further alkylated with 50 mM of iodoacetamide. It was further digested with trypsin (1:50, trypsin/lysate ratio) for 16 h at 37°C. Digests were cleaned using a C18 silica cartridge to remove the salt and dried using a speed vac. The dried pellet was resuspended in buffer A (2% acetonitrile, 0.1% formic acid).

### 2.5 Nano LC-MS/MS

All the experiments were performed quantitatively using the RSLC Nano system (Thermo Fisher Scientific, USA) coupled to Thermo Fisher-*QExactive* Plus equipped with a nano electrospray ion source. One microgram was loaded on a C18 column 50 cm, 3.0 μm EASY-Spray column (Thermo Fisher Scientific). Peptides were eluted with a 0%–40% gradient of buffer B (80% acetonitrile, 0.1% formic acid) at a flow rate of 300 nl/min and injected for mass spectrometry analysis. LC gradients were run for 100 min. MS1 spectra were acquired in the Orbitrap at 70,000 resolution. Dynamic exclusion was employed during data-dependent acquisition (DDA) for 10 s excluding all charge states for a given precursor. This method was chosen to ensure that each fragment obtained can be related back to a specific precursor, as the resulting MS/MS spectrum contains only fragments from the precursor. Moreover, at the same pressure of He gas, the ion trap performs both ion collection and fragmentation in the used dynamic exclusion method. MS2 spectra were acquired at 17,500 resolution. All the processed samples were subjected to mass spectrometry run in technical replicates. Raw files containing mass/charge values were produced for each of the samples. Then, these raw files were analyzed through Thermo Proteome Discoverer (v2.2) against the UniProt Human database. For the SEQUEST and Amanda search, the precursor and fragment mass tolerance were set at 10 ppm and 0.5 Da, respectively. The protease used to generate peptides, i.e., enzyme specificity, was set for trypsin/P (cleavage at the C terminus of “K/R: unless followed by “P”) along with a maximum missed cleavage value of 2. Carbamidomethyl on cysteine was considered as fixed modification, while oxidation of methionine and acetylation of N terminus were both considered as variable modifications for database search. Both peptide spectrum match and protein false discovery rate were set to 0.01 false discovery rate (FDR). Based on UniProt accession number, Pfam, KEGG pathways, and GO annotations were assigned for the list of identified proteins. Differentially expressed proteins (DEPs) were identified by calculating their abundance ratio (treated/untreated) as log2 of fold change.

### 2.6 Bioinformatics analysis

#### 2.6.1 Selection of signature differentially expressed proteins

A web-based gene expression database called the Gene Expression database of Normal and Tumor tissues 2 (GENT2) (http://gent2.appex.kr/gent2/) generated by the Affymetrix U133A or U133Plus2 microarray platform [GPL570 platform (HG-U133_Plus_2)] was utilized to find out the signature secretory proteins out of the total identified DEPs from NVA-AA-treated MCF-7 and MDA-MB-231 cell lines (25). Briefly, the tissue-wide expression pattern of each identified DEP was studied in normal and breast cancer patient samples (*N* = 1,246) to filter out the most important key proteins (DEPs) affected by the treatment of MCF-7 and MDA-MB-231 cells in comparison to untreated cell lines. Two-sample *t*-tests were applied, and filtered-out proteins were further verified for their distinct expression pattern across different cancer subtypes varying in estrogen receptor (ER) status. Similarly, survival analysis for the key proteins was further carried out, and Kaplan–Meier (KM) plots with median cutoff were produced to investigate the impact of their gene expression on overall survival (OS) of breast cancer patients, as well as their clinical significance and prognostic value. As MCF-7 and MDA-MB-231 represent luminal A and triple-negative breast cancer (TNBC) subtypes, respectively, therefore, the identified key proteins from MCF-7 and MDA-MB-231 were further compared with the existing expression profile across luminal A and TNBC subtype using GENT2. *P*-value <0.05 was considered statistically significant for expression as well as survival analysis.

#### 2.6.2 Correlation of gene expression with drug sensitivity and immune cell infiltration in GSCA

An integrated platform, Gene Set Cancer Analysis (GSCA) (http://bioinfo.life.hust.edu.cn/GSCA/#/) (26), was used for Pearson correlation analysis between mRNA expression of selected signature genes and drug sensitivity (IC_50_) of 265 small molecules and 481 compounds toward breast cancer, provided by the Genomics of Drug Sensitivity in Cancer (GDSC) (https://www.cancerrxgene.org/) and Cancer Therapeutics Response Portal (CTRP) (https://portals.broadinstitute.org/ctrp/) datasets, respectively. *P*-value was adjusted by FDR. Red and blue colors specify positive and negative correlations, respectively, such that a darker color represents a stronger correlation (*: *P*-value < 0.05; #: FRD < 0.05). These analyses would help to predict the sensitivity of MCF-7 and MDA-MB-231 toward NVA-AA NP treatments, which are shared with the list of anticancer drugs provided by the GDSC and CTRP. A positive correlation indicates that a high expression of genes provides a high resistance toward the drug and vice versa.

GSCA also provides multidimensional genomic data across 33 cancer types from the Cancer Genome Atlas (TCGA). Spearman correlation analysis was applied to estimate the association between signature gene mRNA expression and 24 immune cell infiltrates in BRCA through the ImmuCellAI module of GSCA (27). Immune infiltration and the GSVA score module of GSCA were utilized to study the correlation between signature gene expression and immune cell infiltrates in the BRCA cancer type. *P*-value was adjusted by FDR. Red and blue colors indicate positive and negative correlations, respectively, with a darker color representing a stronger correlation (*: *P*-value < 0.05; #: FDR < 0.05).

#### 2.6.3 Gene annotation and KEGG analysis by GeneCodis

Singular and modular enrichment analysis (SEA and MEA) of the selected signature DEPs was done using an online web tool, GeneCodis (https://genecodis.genyo.es/). This tool uses standard hypergeometric or Wallenius’ non-central hypergeometric distribution for enrichment analysis, where hypergeometric or Wallenius tested the raw *P*-values and corrected them *via* Benjamini/Hochberg FDR. Different modules, namely, KEGG, Reactome, gene annotation for cellular components, PharmGKB, and DisGeNET, were used to determine the association of these signature genes with other metabolic pathways, cellular components, pharmacogenomics information about drug responses, and other human diseases, respectively. The bar charts were generated, where bar size signifies −log10(Pval Adj) and the red intensity signifies the number of genes present (28).

#### 2.6.4 Protein–protein interaction and PANTHER analysis

The protein–protein interaction (PPI) among significantly differentially expressed signature proteins selected after nano LC-MS/MS analysis was built using the STRING module of Cytoscape version 3.7.1. Proteins of interest were enlisted into the STRING database using their UniProt IDs to generate the interaction between these proteins to highlight the mechanism involved in cytotoxicity caused by NVA-AA treatment and identify secretory protein targets. All the identified proteins were also classified based on their molecular function, biological roles, pathways, cellular components, and protein classes using PANTHER 17.0 (http://www.pantherdb.org/) online software.

### 2.7 Real-time PCR

Validation of the expression of selected altered signature DEPs, namely, gelsolin (GSN) (actin-binding protein and tumor suppressor), complement component C3 (C3) (component of innate immune response), and cytochrome c somatic (CYCS) (apoptotic biomarker) due to NVA-AA treatment of MCF-7 and MDA-MB-231 cell lines, was done by quantitation of the corresponding mRNA transcripts using quantitative PCR. The total RNA from the untreated and treated MCF-7 and MDA-MB-231 cell lines was isolated using the RNASure^®^ Fusion RNA Mini Kit. The quantity and quality of RNA were assessed using NanoDrop 2000c spectrophotometer. The cDNA synthesis was done using normalized RNA samples by RevertAid H Minus Reverse Transcriptase (Thermo Scientific) Kit. Quantitative PCR of synthesized cDNA was performed on the Bio-Rad real-time PCR cycler using SYBR Green chemistry. The total reaction volume was made 10 μl which included 5 μl of 2× Fermentas Maxima SYBR Green qPCR Master Mix (Thermo Scientific), 0.25 μl of each primer (20 μM) (**Supplementary Table S1**), 2 μl of cDNA, and 2.5 μl of nuclease-free water. All reactions were performed in triplicates with the following temperature conditions: i) 96°C for 5 min (initial denaturation) and ii) 34 cycles at 96°C for 1 min, 56°C for 1 min, and 72°C for 5 min, followed by iii) melt curve analysis at a temperature range of 55°C–95°C. The raw data were analyzed with the CFX Maestro software (version 2.3). The Ct values were obtained using a constant threshold value for all the genes assayed. The relative gene expression was quantitated by applying a comparative ΔCt (2^−ΔΔCT^) method. The untreated sample was used as the calibrator/control. HPRT1 served as the normalizer gene.

### 2.8 Statistical analysis

Data were analyzed through unpaired *t*-test for the identification of differentially expressed secretory proteins among NVA-AA NP-treated and untreated MCF-7 and MDA-MB-231 cell lines using GraphPad Prism software (version 7.04, GraphPad software). *P*-value <0.05 was considered statistically significant.

## 3 Results

In this study, polymeric nanoparticles were synthesized using NIPAAM, VP, and AA with the average size of 131.4 nm ± 19.7 nm (by DLS) and 110 nm ± 12.6 nm (by TEM) (19). *Artemisia absinthium* ethanolic extract was encapsulated in the hydrophobic core of these polymeric nanoparticles (NVA-AA NPs). Nano LC-MS/MS was employed to quantify the unique proteins secreted in the media of NVA-AA-treated MCF-7 and MDA-MB-231 cell lines. Raw data files obtained from nano LC-MS/MS were analyzed against the human subset of the RefSeq database from NCBI, and the master list of proteins was prepared with their relative quantification. Unique proteins were selected out of the total protein groups and peptide groups identified in Thermo Proteome Discoverer against the Human database based on abundance count (=1). Among these, 65 and 156 proteins were significantly differentially expressed (*P* < 0.05) in MCF-7 and MDA-MB-231 cell lines (as shown in **Supplementary Figures S1A, B**), respectively. The lists of a few important significant DEPs in MCF-7 and MDA-MB-231 are shown in **Tables 1**, **2**, respectively.

**Table 1:** List of some significantly differentially expressed proteins in NVA-AA NP-treated MCF-7 cell line with their abundance ratio and abundance ratio (log2).

Accession no.	Protein name	Gene	Abundance ratio: (MCF-7 treatment)/(control MCF-7)	Abundance ratio (log2): (MCF-7 treatment)/(control MCF-7)
Q05639	Elongation factor 1-alpha 2	EEF1A2	0.55212813	−0.85692499
P68104	Elongation factor 1-alpha 1	EEF1A1	0.669922678	−0.5779335
Q5VTE0	Putative elongation factor 1-alpha-like 3	EEF1A1P5	0.669922678	−0.5779335
P57053	Histone H2B type F-S	H2BFS	1.483903394	0.56939717
P23527	Histone H2B type 1-O	HIST1H2BO	1.483903394	0.56939717
P06899	Histone H2B type 1-J	HIST1H2BJ	1.483903394	0.56939717
Q5QNW6	Histone H2B type 2-F	HIST2H2BF	1.483903394	0.56939717
Q93079	Histone H2B type 1-H	HIST1H2BH	1.483903394	0.56939717
P33778	Histone H2B type 1-B	HIST1H2BB	1.483903394	0.56939717
Q96A08	Histone H2B type 1-A	HIST1H2BA	1.483903394	0.56939717
P62807	Histone H2B type 1-C/E/F/G/I	HIST1H2BC	1.483903394	0.56939717
Q99880	Histone H2B type 1-L	HIST1H2BL	1.483903394	0.56939717
Q16778	Histone H2B type 2-E	HIST2H2BE	1.483903394	0.56939717
Q8N257	Histone H2B type 3-B	HIST3H2BB	1.483903394	0.56939717
Q99879	Histone H2B type 1-M	HIST1H2BM	1.483903394	0.56939717
Q99877	Histone H2B type 1-N	HIST1H2BN	1.483903394	0.56939717
O60814	Histone H2B type 1-K	HIST1H2BK	1.483903394	0.56939717
P58876	Histone H2B type 1-D	HIST1H2BD	1.483903394	0.56939717
P99999	Cytochrome c	CYCS	3.813895941	1.93126548
P62805	Histone H4	HIST1H4A	5.92928535	2.56785823
P31150	Rab GDP dissociation inhibitor alpha	GDI1	7.200903224	2.84817788
P50395	Rab GDP dissociation inhibitor beta	GDI2	7.200903224	2.84817788
P20671	Histone H2A type 1-D	HIST1H2AD	11.18755836	3.4838233
Q96KK5	Histone H2A type 1-H	HIST1H2AH	11.18755836	3.4838233
Q7L7L0	Histone H2A type 3	HIST3H2A	11.18755836	3.4838233
P04908	Histone H2A type 1-B/E	HIST1H2AB	11.18755836	3.4838233
Q9BTM1	Histone H2A.J	H2AFJ	11.18755836	3.4838233
Q99878	Histone H2A type 1-J	HIST1H2AJ	11.18755836	3.4838233
P0C0S8	Histone H2A type 1	HIST1H2AG	11.18755836	3.4838233
Q93077	Histone H2A type 1-C	HIST1H2AC	11.18755836	3.4838233
Q8IUE6	Histone H2A type 2-B	HIST2H2AB	11.49650496	3.52312343
P16104	Histone H2AX	H2AFX	11.49650496	3.52312343
Q5T7W0	Zinc finger protein 618	ZNF618	11.78912112	3.55938426
P60709	Actin, cytoplasmic 1	ACTB	17.24132959	4.10779913
P63261	Actin, cytoplasmic 2	ACTG1	17.24132959	4.10779913
Q6FI13	Histone H2A type 2-A	HIST2H2AA3	24.71857381	4.6275236
Q16777	Histone H2A type 2-C	HIST2H2AC	24.71857381	4.6275236
P15531	Nucleoside diphosphate kinase A	NME1	32.45804769	5.02050432
Q71UI9	Histone H2A.V	H2AFV	62.08942144	5.95627558
Q96QV6	Histone H2A type 1-A	HIST1H2AA	62.08942144	5.95627558
P0C0S5	Histone H2A.Z	H2AFZ	62.08942144	5.95627558
P07437	Tubulin beta chain	TUBB	62.43938988	5.96438454
P36955	Pigment epithelium-derived factor	SERPINF1	70.27863188	6.1350142
Q99536	Synaptic vesicle membrane protein VAT-1 homolog	VAT1	70.59594493	6.14151341
Q9NY65	Tubulin alpha-8 chain	TUBA8	90.39290674	6.49813766
Q9H853	Putative tubulin-like protein alpha-4B	TUBA4B	90.39290674	6.49813766
P68366	Tubulin alpha-4A chain	TUBA4A	96.04706298	6.58566959
P02771	Alpha-fetoprotein	AFP	96.73526934	6.59597008
P01024	Complement C3	C3	99.1797056	6.63197304
Q06033	Inter-alpha-trypsin inhibitor heavy chain H3	ITIH3	107.9851289	6.75468884
P68363	Tubulin alpha-1B chain	TUBA1B	117.4742599	6.87620087
Q9BQE3	Tubulin alpha-1C chain	TUBA1C	117.4742599	6.87620087
O95497	Pantetheinase	VNN1	118.2435649	6.88561786
P06396	Gelsolin	GSN	165.4226416	7.3700129
P62937	Peptidyl-prolyl cis-trans isomerase A	PPIA	183.678932	7.52104235
P19823	Inter-alpha-trypsin inhibitor heavy chain H2	ITIH2	240.6207154	7.91061704
P02774	Vitamin D-binding protein	GC	328.7863319	8.36100652
P07996	Thrombospondin-1	THBS1	364.7256544	8.51066787
P04264	Keratin, type II cytoskeletal 1	KRT1	391.0226133	8.61110823
P07477	Trypsin-1	PRSS1	630.3362025	9.29997771
P01008	Antithrombin-III	SERPINC1	1,465.479717	10.5171573
P35908	Keratin, type II cytoskeletal 2 epidermal	KRT2	2,230.390675	11.1230807
P02768	Serum albumin	ALB	2,857.264536	11.4804189
P02788	Lactotransferrin	LTF	8,468.862509	13.0479525

**Table 2:** List of some significantly differentially expressed proteins in NVA-AA NP-treated MDA-MB-231 cell line with their abundance ratio and abundance ratio (log2).

Accession no.	Protein name	Gene	Abundance ratio: (MDA-MB-231 treatment)/(control MDA-MB-231)	Abundance ratio (log2): (MDA-MB-231 treatment)/(control MDA-MB-231)
P08238	Heat shock protein HSP 90-beta	HSP90AB1	0.033210773	−4.9122049
P13645	Keratin, type I cytoskeletal 10	KRT10	0.11048254	−3.1781097
P07477	Trypsin-1	PRSS1	0.118042609	−3.0826204
Q58FF7	Putative heat shock protein HSP 90-beta-3	HSP90AB3P	0.172726201	−2.5334412
P68363	Tubulin alpha-1B chain	TUBA1B	0.192955315	−2.3736613
Q9BQE3	Tubulin alpha-1C chain	TUBA1C	0.192955315	−2.3736613
P35908	Keratin, type II cytoskeletal 2 epidermal	KRT2	0.198770368	−2.3308254
P35527	Keratin, type I cytoskeletal 9	KRT9	0.213029276	−2.2308764
P68366	Tubulin alpha-4A chain	TUBA4A	0.224120446	−2.1576538
Q7Z794	Keratin, type II cytoskeletal 1b	KRT77	0.224947674	−2.1523386
P04264	Keratin, type II cytoskeletal 1	KRT1	0.24767609	−2.0134735
P07437	Tubulin beta chain	TUBB	0.26677046	−1.9063292
Q9UK55	Protein Z-dependent protease inhibitor	SERPINA10	0.297543726	−1.7488264
P57053	Histone H2B type F-S	H2BFS	0.323611545	−1.627665
P23527	Histone H2B type 1-O	HIST1H2BO	0.323611545	−1.627665
P06899	Histone H2B type 1-J	HIST1H2BJ	0.323611545	−1.627665
Q5QNW6	Histone H2B type 2-F	HIST2H2BF	0.323611545	−1.627665
Q93079	Histone H2B type 1-H	HIST1H2BH	0.323611545	−1.627665
P33778	Histone H2B type 1-B	HIST1H2BB	0.323611545	−1.627665
Q96A08	Histone H2B type 1-A	HIST1H2BA	0.323611545	−1.627665
P62807	Histone H2B type 1-C/E/F/G/I	HIST1H2BC	0.323611545	−1.627665
Q99880	Histone H2B type 1-L	HIST1H2BL	0.323611545	−1.627665
Q16778	Histone H2B type 2-E	HIST2H2BE	0.323611545	−1.627665
Q8N257	Histone H2B type 3-B	HIST3H2BB	0.323611545	−1.627665
Q99879	Histone H2B type 1-M	HIST1H2BM	0.323611545	−1.627665
Q99877	Histone H2B type 1-N	HIST1H2BN	0.323611545	−1.627665
O60814	Histone H2B type 1-K	HIST1H2BK	0.323611545	−1.627665
P58876	Histone H2B type 1-D	HIST1H2BD	0.323611545	−1.627665
Q5XKE5	Keratin, type II cytoskeletal 79	KRT79	0.401826019	−1.3153571
P68104	Elongation factor 1-alpha 1	EEF1A1	0.444543649	−1.169603
Q5VTE0	Putative elongation factor 1-alpha-like 3	EEF1A1P5	0.444543649	−1.169603
P18669	Phosphoglycerate mutase 1	PGAM1	0.455592927	−1.1341827
P15259	Phosphoglycerate mutase 2	PGAM2	0.455592927	−1.1341827
Q05639	Elongation factor 1-alpha 2	EEF1A2	0.45659995	−1.1309974
P01008	Antithrombin-III	SERPINC1	0.467479405	−1.0970253
Q12767	Transmembrane protein 94	TMEM94	0.482815647	−1.0504557
Q13885	Tubulin beta-2A chain	TUBB2A	0.486568861	−1.0392841
Q9BVA1	Tubulin beta-2B chain	TUBB2B	0.486568861	−1.0392841
Q9BUF5	Tubulin beta-6 chain	TUBB6	0.486568861	−1.0392841
A6NNZ2	Tubulin beta-8 chain-like protein LOC260334		0.486568861	−1.0392841
Q3ZCM7	Tubulin beta-8 chain	TUBB8	0.486568861	−1.0392841
P02008	Hemoglobin subunit zeta	HBZ	0.50396187	−0.988613513
Q12805	EGF-containing fibulin-like extracellular matrix protein 1	EFEMP1	0.516042589	−0.954438
Q71UI9	Histone H2A.V	H2AFV	0.561095947	−0.8336806
Q96QV6	Histone H2A type 1-A	HIST1H2AA	0.561095947	−0.8336806
P0C0S5	Histone H2A.Z	H2AFZ	0.561095947	−0.8336806
Q8IUE6	Histone H2A type 2-B	HIST2H2AB	0.575456734	−0.7972206
P16104	Histone H2AX	H2AFX	0.575456734	−0.7972206
P62805	Histone H4	HIST1H4A	0.593776341	−0.7520085
P05546	Heparin cofactor 2	SERPIND1	0.604385972	−0.7264579
Q99536	Synaptic vesicle membrane protein VAT-1 homolog	VAT1	0.611837782	−0.7087789
Q9HDC9	Adipocyte plasma membrane-associated protein	APMAP	0.629737372	−0.6671778
P62987	Ubiquitin-60S ribosomal protein L40	UBA52	0.639526004	−0.6449251
P0CG48	Polyubiquitin-C	UBC	0.639526004	−0.6449251
P0CG47	Polyubiquitin-B	UBB	0.639526004	−0.6449251
P62979	Ubiquitin-40S ribosomal protein S27a	RPS27A	0.639526004	−0.6449251
P00734	Prothrombin	F2	0.6443508	−0.6340818
O95497	Pantetheinase	VNN1	0.64999062	−0.6215092
O14556	Glyceraldehyde-3-phosphate dehydrogenase, testis-specific	GAPDHS	0.65376806	−0.6131492
P54652	Heat shock-related 70 kDa protein 2	HSPA2	0.662484325	−0.5940418
P02753	Retinol-binding protein 4	RBP4	0.664752847	−0.58911
P02647	Apolipoprotein A-I	APOA1	0.670050531	−0.5776582
P02749	Beta-2-glycoprotein 1	APOH	0.720783147	−0.4723628
Q13162	Peroxiredoxin-4	PRDX4	0.746819494	−0.4211685
P01031	Complement C5	C5	0.756197821	−0.4031644
P20671	Histone H2A type 1-D	HIST1H2AD	0.757068061	−0.4015051
Q96KK5	Histone H2A type 1-H	HIST1H2AH	0.757068061	−0.4015051
Q7L7L0	Histone H2A type 3	HIST3H2A	0.757068061	−0.4015051
P04908	Histone H2A type 1-B/E	HIST1H2AB	0.757068061	−0.4015051
Q9BTM1	Histone H2A.J	H2AFJ	0.757068061	−0.4015051
Q99878	Histone H2A type 1-J	HIST1H2AJ	0.757068061	−0.4015051
P0C0S8	Histone H2A type 1	HIST1H2AG	0.757068061	−0.4015051
Q93077	Histone H2A type 1-C	HIST1H2AC	0.757068061	−0.4015051
Q6FI13	Histone H2A type 2-A	HIST2H2AA3	0.762822329	−0.390581
Q16777	Histone H2A type 2-C	HIST2H2AC	0.762822329	−0.390581
P07996	Thrombospondin-1	THBS1	0.796052856	−0.3290639
P0C0L5	Complement C4-B	C4B	0.800578769	−0.3208847
Q9H4B7	Tubulin beta-1 chain	TUBB1	0.80524181	−0.312506
P62826	GTP-binding nuclear protein Ran	RAN	0.812284881	−0.2999423
P05543	Thyroxine-binding globulin	SERPINA7	0.813257408	−0.298216
P20742	Pregnancy zone protein	PZP	0.81417467	−0.2965898
P11142	Heat shock cognate 71 kDa protein	HSPA8	0.834245928	−0.2614554
P07864	L-lactate dehydrogenase C chain	LDHC	0.839311485	−0.2527218
P19827	Inter-alpha-trypsin inhibitor heavy chain H1	ITIH1	0.839988426	−0.2515586
P00751	Complement factor B	CFB	0.854893171	−0.2261839
P55058	Phospholipid transfer protein	PLTP	0.880183581	−0.1841236
P01023	Alpha-2-macroglobulin	A2M	0.881286505	−0.182317
P04114	Apolipoprotein B-100	APOB	0.893138952	−0.1630435
P05452	Tetranectin	CLEC3B	0.912483501	−0.1321296
P02774	Vitamin D-binding protein	GC	0.923639034	−0.114599
P99999	Cytochrome c	CYCS	0.940075116	−0.0891521
P02768	Serum albumin	ALB	0.959795363	−0.059201251
P0C0L4	Complement C4-A	C4A	0.978613676	−0.0311887
P02788	Lactotransferrin	LTF	1.021491015	0.0306765
P18065	Insulin-like growth factor-binding protein 2	IGFBP2	1.025299299	0.0360451
P02649	Apolipoprotein E	APOE	1.033014967	0.0468612
P12109	Collagen alpha-1(VI) chain	COL6A1	1.067208398	0.0938419
Q8NBM4	Ubiquitin-associated domain-containing protein 2	UBAC2	1.069573599	0.0970358
P19823	Inter-alpha-trypsin inhibitor heavy chain H2	ITIH2	1.101386466	0.1393208
P49747	Cartilage oligomeric matrix protein	COMP	1.121033574	0.1648295
P34931	Heat shock 70 kDa protein 1-like	HSPA1L	1.13344724	0.1807172
P0DMV9	Heat shock 70 kDa protein 1B	HSPA1B	1.13344724	0.1807172
P48741	Putative heat shock 70 kDa protein 7	HSPA7	1.13344724	0.1807172
P17066	Heat shock 70 kDa protein 6	HSPA6	1.13344724	0.1807172
P01042	Kininogen 1	KNG1	1.140047283	0.1890937
P06733	Alpha-enolase	ENO1	1.147120827	0.1980174
Q5T7W0	Zinc finger protein 618	ZNF618	1.170760381	0.2274458
P02765	Alpha-2-HS-glycoprotein	AHSG	1.186172253	0.2463135
Q04756	Hepatocyte growth factor activator	HGFAC	1.238047016	0.3080661
P09486	SPARC	SPARC	1.256863148	0.3298276
P10643	Complement component C7	C7	1.273610642	0.3489243
P01024	Complement C3	C3	1.282217663	0.3586412
P02751	Fibronectin	FN1	1.291127547	0.3686315
P02452	Collagen alpha-1(I) chain	COL1A1	1.297688573	0.3759442
P02771	Alpha-fetoprotein	AFP	1.306425823	0.3856252
Q06033	Inter-alpha-trypsin inhibitor heavy chain H3	ITIH3	1.327071557	0.4082462
P02042	Hemoglobin subunit delta	HBD	1.374183746	0.458574924
P68871	Hemoglobin subunit beta	HBB	1.374183746	0.458574924
Q71U36	Tubulin alpha-1A chain	TUBA1A	1.387615881	0.4726083
P13929	Beta-enolase	ENO3	1.422720431	0.5086522
P09104	Gamma-enolase	ENO2	1.422720431	0.5086522
P06396	Gelsolin	GSN	1.422833088	0.5087664
P69891	Hemoglobin subunit gamma-1	HBG1	1.462791445	0.548724094
P69892	Hemoglobin subunit gamma-2	HBG2	1.462791445	0.548724094
P02100	Hemoglobin subunit epsilon	HBE1	1.462791445	0.548724094
P23142	Fibulin-1	FBLN1	1.507190543	0.5918618
P04745	Alpha-amylase 1	AMY1A	1.51107115	0.5955716
P19961	Alpha-amylase 2B	AMY2B	1.51107115	0.5955716
P04746	Pancreatic alpha-amylase	AMY2A	1.51107115	0.5955716
P36955	Pigment epithelium-derived factor	SERPINF1	1.585904488	0.6653059
Q6ZMR3	L-lactate dehydrogenase A-like 6A	LDHAL6A	1.602294139	0.680139
P07195	L-lactate dehydrogenase B chain	LDHB	1.602294139	0.680139
Q14515	SPARC-like protein 1	SPARCL1	1.610638335	0.6876326
P02545	Prelamin-A/C	LMNA	1.623443781	0.6990574
P00338	L-lactate dehydrogenase A chain	LDHA	1.624785099	0.7002489
P15169	Carboxypeptidase N catalytic chain	CPN1	1.632362261	0.7069613
P60709	Actin, cytoplasmic 1	ACTB	1.643385654	0.7166711
P63261	Actin, cytoplasmic 2	ACTG1	1.643385654	0.7166711
P04406	Glyceraldehyde-3-phosphate dehydrogenase	GAPDH	1.679349457	0.7479025
P05156	Complement factor I	CFI	1.803620716	0.850896
P00747	Plasminogen	PLG	1.838993584	0.8789164
Q15063	Periostin	POSTN	1.854076965	0.8907011
P04004	Vitronectin	VTN	1.874376642	0.9064109
Q01546	Keratin, type II cytoskeletal 2 oral	KRT76	1.960202979	0.9710031
P19013	Keratin, type II cytoskeletal 4	KRT4	1.960202979	0.9710031
P69905	Hemoglobin subunit alpha	HBA1	2.085726024	1.060549661
P02748	Complement component C9	C9	2.276700387	1.1869444
P51884	Lumican	LUM	2.283541957	1.1912733
Q9UN73	Protocadherin alpha-6	PCDHA6	2.975608641	1.5731848
P14618	Pyruvate kinase PKM	PKM	3.209592842	1.6823903
P50395	Rab GDP dissociation inhibitor beta	GDI2	3.970767406	1.9894179
P31150	Rab GDP dissociation inhibitor alpha	GDI1	3.970767406	1.9894179
P63104	14-3-3 protein zeta/delta	YWHAZ	5.077837083	2.3442141
P08697	Alpha-2-antiplasmin	SERPINF2	12.32952965	3.6240459
P61978	Heterogeneous nuclear ribonucleoprotein K	HNRNPK	14.29246859	3.8371832

### 3.1 Secretome profiling of MCF-7 and MDA-MB-231 after NVA-AA NP treatment

A comparative analysis of the untreated and NVA-AA-treated MCF-7 and MDA-MB-231 cell lines revealed alteration in key secretory proteins involved in apoptosis, cell proliferation, tumorigenesis, metastasis, cancer progression, and suppression. In MCF-7, out of 65 proteins, some of the upregulated proteins (fold change ≥ 1) are involved in apoptosis, e.g., cytochrome c (CYCS), histone H2AX (H2AFX), nucleoside diphosphate kinase A (NME1), and lactotransferrin (LTF); a few are involved in vesicular trafficking, e.g., Rab GDP dissociation inhibitor beta (GDI2), while others are involved in the inhibition of angiogenesis, e.g., pigment epithelium-derived factor (SERPINF1) and thrombospondin-1 (THBS1). Some tumor suppressor genes are also highly upregulated in NVA-AA-treated cells, e.g., inter-alpha-trypsin inhibitor heavy chain H3 (ITIH3) and gelsolin (GSN), while proteins involved in the inhibition of cell proliferation and metastasis are also upregulated, e.g., vitamin D-binding protein (GC). However, a few proteins involved in tumorigenesis were downregulated (fold change ≤ −1), e.g., elongation factor 1-alpha 2 (EEF1A2) and elongation factor 1-alpha 1 (EEF1A1). A few proteins belonging to the histone family (HIST1H4A, HIST1H2AD, H2AFX, HIST2H2AC, H2AFZ) had upregulated expression, suggesting that NVA-AA NPs affected the genome integrity of treated MCF-7 cells through nucleosome disassembly as compared with untreated cells. Increased expression of tubulin isoforms (TUBB, TUBA8, TUBA4A, TUBA1B) indicates the potential of NVA-AA to hamper the dynamics of microtubule assembly in MCF-7 cells. This could be linked with the downregulation of EEF1A2 and EEF1A1 in the treated cells that are required for cytoskeletal protein rearrangement comprised of actin and tubulins.

Similarly, proteins involved in the inhibition of cancer invasion are highly upregulated (fold change ≥ 1) in NVA-AA-treated MDA-MB-231 cells, e.g., lumican (LUM), along with proteins involved in vesicular trafficking, e.g., Rab GDP dissociation inhibitor beta (GDI2). Moreover, proteins involved in proliferation and tumorigenesis were downregulated (fold change ≤ −1) after treatment, e.g., elongation factor 1-alpha 1 (EEF1A1), elongation factor 1-alpha 2 (EEF1A2), peroxiredoxin-4 (PRDX4), GTP-binding nuclear protein Ran (RAN), and alpha-enolase (ENO1), as well as proteins involved in proteosomal degradation, cell adhesion, and migration, e.g., ubiquitin-60S ribosomal protein L40 (UBA52), fibronectin (FN1), and periostin (POSTN). The downregulation of tubulin family genes, TUBA1B, TUBA4A, TUBB, and TUBB8, after NVA-AA NP treatment in MDA-MB-231 suggests a disturbance in tubulin assembly. Heat shock protein HSP 90-beta (HSP90AB1), which is associated with the survival of certain oncogenes, was also significantly downregulated by NVA-AA NPs. Proteins belonging to the family of keratins (KRT1, KRT10, KRT2, KRT77) were also downregulated in our study.

### 3.2 Bioinformatic analysis

#### 3.2.1 Identification of signature DEPs

**Figure 1:**
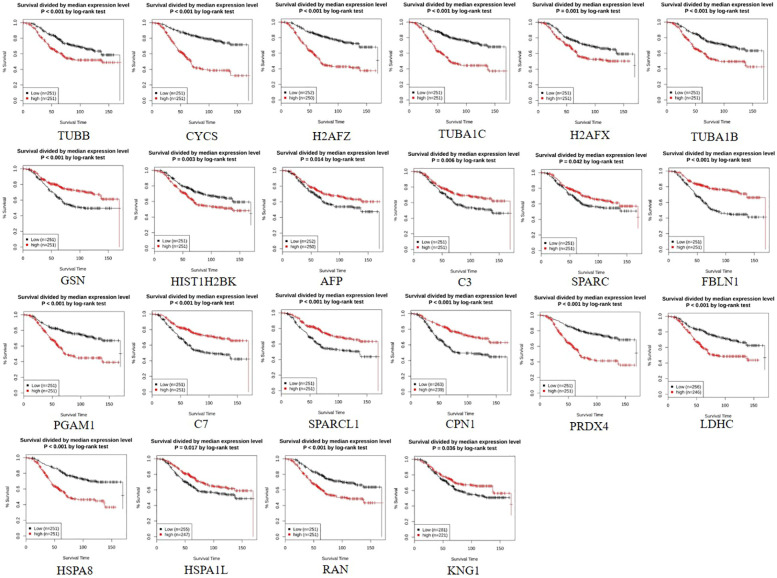
Survival curve of 22 signature differentially expressed proteins (DEPs) obtained from N-isopropyl acrylamide, N-vinyl pyrrolidone, and acrylic acid-based polymeric nanoparticle (NVA-AA NP)-treated MCF-7 and MDA-MB-231 cell lines showing their clinical significance and prognostic value in breast cancer as generated by GENT2 employed for overall survival (OS) analysis. The low expression of TUBB, CYCS, H2AFZ, TUBA1C, H2AFX, TUBA1B, HIST1H2BK, PGAM1, PRDX4, LDHC, HSPA8, and RAN, which are considered favorable for prognosis in breast cancer patients, coincides with their downregulation in NVA-AA NP-treated cell lines indicating a favorable outcome of NVA-AA NP treatment. Similarly, the high expression of GSN, AFP, C3, SPARC, FBLN1, C7, SPARCL1, CPN1, HSPA1L, and KNG1 correlates with their upregulation in NVA-AA NP-treated cell lines and justifies the favorable outcome of the treatment.

Out of the total proteins, analysis by GENT2 revealed 22 signature DEPs having the most significant change in their expression level after NVA-AA treatment of MCF-7 and MDA-MB-231. Differential expression levels, fold change (log2FC), and the *P*-values of these proteins in breast cancer tissues are represented along with their expression after treatment in the affected cell lines in **Table 3**. The expression profile of selected signature DEPs across cancer subtypes differing in ER status is represented in **Supplementary Figure S2** and their survival analysis is represented in **Figure 1**. Fold change and the expression profile of suitable signature genes from MCF-7 and MDA-MB-231 were again compared across luminal A and TNBC subtypes as shown in **Supplementary Figure S3** and **Supplementary Table S2**.

**Table 3:** List of 22 signature differentially expressed proteins (DEPs) in NVA-AA NP-treated MCF-7 and MDA-MB-231 cell lines obtained from the analysis by the GENT2 online database whose expression was found to be most altered.

S. no.	DEP	Fold change in breast cancer tissue	*P*-value	Expressed in the cell line	Expression after treatment
1	Tubulin beta chain (TUBB)	0.219	<0.001	MCF-7; MDA-MB-231	Downregulated in MDA-MB-231
2	Cytochrome C somatic (CYCS)	0.304	<0.001	MCF-7; MDA-MB-231	Upregulated in MCF-7; downregulated in MDA-MB-231
3	Histone H2A.Z (H2AFZ)	0.813	<0.001	MCF-7; MDA-MB-231	Downregulated in MDA-MB-231
4	Tubulin alpha-1C chain (TUBA1C)	0.161	<0.001	MCF-7; MDA-MB-231	Downregulated in MDA-MB-231
5	Histone H2AFX (H2AFX)	0.895	<0.001	MCF-7; MDA-MB-231	Downregulated in MDA-MB-231
6	Tubulin alpha-1B chain (TUBA1B)	0.154	<0.001	MCF-7; MDA-MB-231	Downregulated in MDA-MB-231
7	Gelsolin (GSN)	−1.564	<0.001	MCF-7; MDA-MB-231	Upregulated in both MCF-7 and MDA-MB-231
8	Histone H2B type 1-K (HIST1H2BK)	1.397	<0.001	MCF-7; MDA-MB-231	Downregulated in both MCF-7 and MDA-MB-231
9	Alpha-fetoprotein (AFP)	−1.151	<0.001	MCF-7; MDA-MB-231	Upregulated in both MCF-7 and MDA-MB-231
10	Complement component C3 (C3)	−0.599	<0.001	MCF-7; MDA-MB-231	Upregulated in both MCF-7 and MDA-MB-231
11	SPARC	−0.192	<0.001	MDA-MB-231	Upregulated in MDA-MB-231
12	Fibulin-1 (FBLN1)	−0.972	<0.001	MDA-MB-231	Upregulated in MDA-MB-231
13	Phosphoglycerate mutase 1 (PGAM1)	0.074	0.046	MDA-MB-231	Downregulated in MDA-MB-231
14	Complement component C7 (C7)	−1.744	<0.001	MDA-MB-231	Upregulated in MDA-MB-231
15	SPARC-like protein 1 (SPARCL1)	−1.036	<0.001	MDA-MB-231	Upregulated in MDA-MB-231
16	Carboxypeptidase N catalytic chain (CPN1)	−0.567	<0.001	MDA-MB-231	Upregulated in MDA-MB-231
17	Peroxiredoxins 4 (PRDX4)	0.287	<0.001	MDA-MB-231	Downregulated in MDA-MB-231
18	Lactate dehydrogenase C (LDHC)	0.178	0.009	MDA-MB-231	Downregulated in MDA-MB-231
19	Heat shock cognate 71 kDa protein (HSPA8)	0.336	<0.001	MDA-MB-231	Downregulated in MDA-MB-231
20	Heat shock 70 kDa protein 1-like (HSPA1L)	−0.14	0.002	MDA-MB-231	Upregulated in MDA-MB-231
21	GTP-binding nuclear protein Ran (RAN)	0.211	<0.001	MDA-MB-231	Downregulated in MDA-MB-231
22	Kininogen 1 (KNG1)	−0.365	<0.001	MDA-MB-231	Upregulated in MDA-MB-231

#### 3.2.2 Correlation analysis between mRNA of signature DEP and drug sensitivity by GSCA

**Figure 2:**
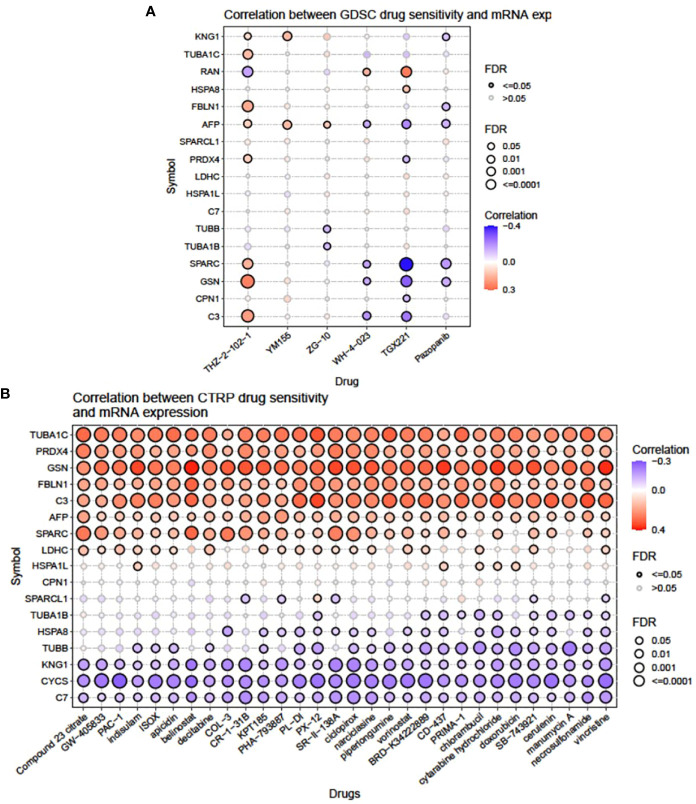
Illustration showing the correlation between mRNA expression of individual signature genes and drug sensitivity from the **(A)** GDSC and **(B)** CTRP portal.

Bubble plots were obtained from GSCA showing the correlation between mRNA of selected signature DEPs and drug response as shown in **Figure 2**. Inputted gene having an association with at least one drug and vice versa was obtained. Bubble size is positively correlated with the FDR significance, where the blue bubble indicates a negative correlation, the red bubble indicates a positive correlation, and the darker color indicates a higher correlation. As per the analysis, out of the 22 signature DEPs, 17 were found to be correlated with 6 GDSC drugs (**Figure 2A**). A CDK7 inhibitor, THZ-2-102-1, was found to be highly positively correlated with the expression of GSN (FDR <= 0.0001) and C3 (FDR = 0.001); moderately positively correlated with FBLN1, TUBA1C, and SPARC (FDR = 0.01); least positively correlated with KNG1, AFP, and PRDX4 (FDR = 0.05); and negatively correlated with RAN (FDR = 0.01). YM155 which is a survivin suppressant showed a positive correlation with KNG1 and AFP (FDR = 0.01). A JNK1 inhibitor, ZG-10, had a positive correlation with AFP (FDR = 0.05) and a negative correlation with TUBB and TUBA1B (FDR = 0.05). WH-4-023, an Lck and Src inhibitor, was positively correlated with RAN (FDR = 0.05) while negatively correlated with AFP, SPARC, GSN, and C3 (FDR = 0.05). A membrane permeability inhibitor of PI3K p110β subunit, TGX221, was highly positively correlated with RAN (FDR = 0.001) and least positively correlated with HSPA8 (FDR = 0.05), while it was highly negatively correlated with SPARC (FDR <= 0.0001), moderately negatively correlated with AFP, GSN, and C3 (FDR = 0.001), and least negatively correlated with PRDX4 and CPN1 (FDR = 0.05). Pazopanib, which is a tyrosine kinase inhibitor, displayed a moderate negative correlation with SPARC (FDR = 0.001) and a low negative correlation with GSN, AFP, FBLN1, and KNG1 (FDR = 0.05).

Thirty CTRP drugs acting as inhibitors of different signaling pathways, namely, compound 23 citrate, GW-405833, PAC-1, indisulam, ISOX, apicidin, belinostat, decitabine, COL-3, CR-1-31B, KPT185, PHA-793887, PL-DI, PX-12, SR-II-138A, ciclopirox, narciclasine, piperlongumine, vorinostat, BRD-K34222889, CD-437, PRIMA-1, chlorambucil, cytarabine hydrochloride, doxorubicin, SB-743921, cerulenin, manumycin A, necrosulfonamide, and vincristine, showed a variable degree of correlation with the mRNA expression of selected signature DEPs (**Figure 2B**). Out of these, TUBA1C, PRDX4, GSN, FBLN1, C3, and AFP exhibited the highest range of positive correlation (FDR <= 0.0001; 0.001) with all the 30 drugs. SPARC, LDHC, and HSPA1L showed moderate to low levels of positive correlation (FDR = 0.01; 0.05) with many drugs. However, KNG1, CYCS, and C7 had the highest negative correlation (FDR <= 0.0001; 0.001) with all the 30 drugs. TUBB, HSPA8, and TUBA1B showed moderate to low levels of negative correlation (FDR = 0.01; 0.05) with many drugs.

#### 3.2.3 Correlation analysis between mRNA expression of signature DEP and immune cell infiltrates by GSCA

**Figure 3:**
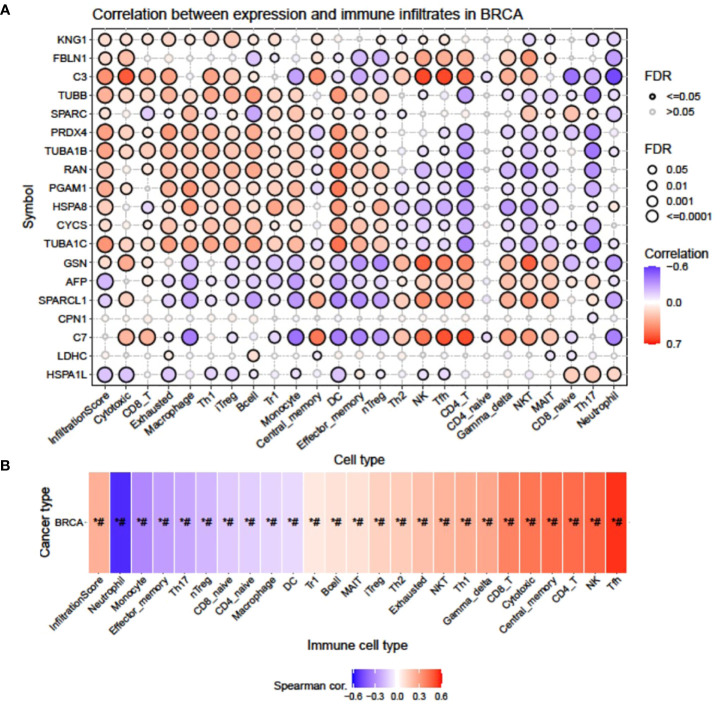
Correlation between mRNA expression of signature DEPs and 24 immune cell infiltrates by GSCA. **(A)** Bubble plot showing the correlation of individual genes with different immune cell infiltrates and **(B)** heatmap showing the cumulative correlation of a set of genes with individual immune cell infiltrates.

A bubble plot was generated indicating a correlation between mRNA expression of signature DEPs and 24 immune cell infiltrates (**Figure 3A**), where C3, TUBB, PRDX4, TUBA1B, RAN, PGAM1, HSPA8, and TUBA1C presented the highest significant positive correlation with infiltration score (FDR <= 0.0001), whereas KNG1, FBLN1, CYCS, GSN, and SPARC had moderate to low positive correlation with infiltration score (FDR = 0.001; 0.01). AFP displayed the highest negative correlation with infiltration score (FDR <= 0.0001), and SPARCL1 and HSPA1L showed moderate and low negative correlation (FDR=0.001; 0.01). Upregulated expression of KNG1 showed a positive correlation with infiltrates of cytotoxic, CD8_T, Exhausted, macrophage, Th1, iTreg, B cell, Tr1, central_memory, NK, and Tfh and a negative correlation with NKT, MAIT, Th17, and neutrophil cell types. The FBLN1-altered expression had the highest positive correlation with cytotoxic, NK, Tfh, CD4_T, Gamma_delta, and NKT and negative correlation with effector_memory, nTreg, and neutrophils. Enhanced expression of C3 in both cell lines correlated positively with cytotoxic, CD8_T, Exhausted, Th1, iTreg, B cell, Th2, NK, Tfh, CD4_T, Gamma_delta, and NKT and negatively with monocyte, CD8_naive, Th17, neutrophil, and CD4_naive. TUBB had a positive correlation with cytotoxic, CD8_T, Exhausted, macrophage, Th1, iTreg, B cell, Tr1, monocyte, DC, effector_memory, and nTreg and a negative correlation with CD4_T, CD4_naive, Gamma_delta, NKT, MAIT, CD8_naive, Th17, and neutrophil. SPARC correlated positively with macrophage, Tr1, monocyte, NKT, and CD8_naive and negatively with B cell and neutrophil. Similarly, a change in the expression of PRDX4, SPARCL1, AFP, GSN, TUBA1C, CYCS, HSPA8, PGAM1, RAN, TUBA1B, C7, and HSPA1L also showed a positive and negative correlation with the maximum number of different infiltrate cell types with a variable degree of FDR.

The heatmap (**Figure 3B**) obtained from the Spearman correlation analysis showed the association of expression of our signature gene set with 24 immune cell infiltrates on the horizontal axis. *P*-value was adjusted by FDR. Red and blue colors indicate positive and negative correlations, respectively, with the darker color representing the stronger correlation (*: *P*-value < 0.05; #: FDR < 0.05). It was observed that the expression pattern of our signature genes was positively correlated (*P*-value ≤ 0.05; #: FDR ≤ 0.05) with Tfh, NK, CD4_T, central_memory, cytotoxic, CD8_T, Gamma_delta, NKT, Th1, Exhausted, Th2, iTreg, MAIT, B cell, and Tr1 and negatively correlated (*P*-value ≤ 0.05; #: FDR ≤ 0.05) with neutrophil, monocyte, effector_memory, Th17, nTreg, CD8_naive, CD4_naive, macrophage, and DC.

#### 3.2.4 Gene annotation and KEGG analysis by GeneCodis

**Figure 4:**
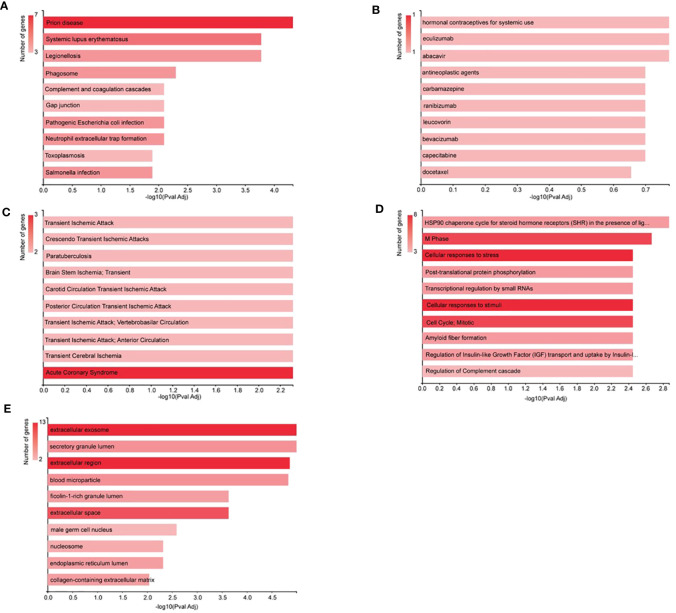
Analysis by GeneCodis. All the signature DEPs from NVA-AA-treated MCF-7 and MDA-MB-231 were categorized by **(A)** KEGG molecular pathways, **(B)** PharmGKB, **(C)** DisGeNET, **(D)** cellular component, and **(E)** Reactome modules.

All the signature DEPs selected in NVA-AA-treated MCF-7 and MDA-MB-231 were categorized based on KEGG molecular pathways, PharmGKB, DisGeNET, cellular component, and Reactome modules. The results of this analysis are shown in **Figure 4**. KEGG enrichment analysis illustrated that these genes were associated with the prion disease (C7, CYCS, HSPA1L, TUBA1B, TUBB), legionellosis (C3, CYCS, HSPA1L, HSPA8), systemic lupus erythematosus (C3, C7, H2AFX, H2AFZ HIST1H2BK), phagosome (C3, TUBA1B, TUBA1C, TUBB), gap junction (C3, TUBA1B, TUBA1C, TUBB), complement and coagulation cascades (KNG1, C3, C7), and neutrophil extracellular trap formation (C3, H2AFX, H2AFZ, HIST1H2BK). PharmGKB analysis revealed an association of these genes with responses toward different drugs, namely, hormonal contraceptives for systemic use (KNG1), eculizumab (C3), abacavir (HSPA1L), antineoplastic agents (SPARC), carbamazepine (HSPA1L), ranibizumab (C3), leucovorin (SPARC), bevacizumab (C3), capecitabine (SPARC), and docetaxel (PRDX4). The DisGeNET module showed the relationship of different signature genes with other human diseases like acute coronary syndrome (HSPA8, GSN, TUBA1C); transient ischemic attack (CYCS, C3); crescendo transient ischemic attack (CYCS, C3); paratuberculosis (C3, GSN); brain stem ischemia, transient (CYCS, C3); carotid circulation transient ischemic attack (CYCS, C3); posterior circulation transient ischemic attack (CYCS, C3), transient ischemic attack; vertebrobasilar circulation (CYCS, C3), transient ischemic attack; anterior circulation (CYCS, C3); and transient cerebral ischemia (CYCS, C3). Reactome analysis by GeneCodis revealed the association of the signature gene with other biological pathways like M phase (H2AFX, H2AFZ, HIST1H2BK, RAN, TUBA1B, TUBA1C, TUBB); cellular responses to stress (CYCS, H2AFX, H2AFZ, HIST1H2BK, HSPA1L, HSPA8, TUBA1B, TUBA1C); cellular responses to stimuli (CYCS, H2AFX, H2AFZ, HIST1H2BK, HSPA1L, HSPA8, TUBA1B, TUBA1C), cell cycle; mitotic (H2AFX, H2AFZ, HIST1H2BK, RAN, TUBA1B, TUBA1C, TUBB); HSP90 chaperone cycle for steroid hormone receptors (SHR) in the presence of ligand (HSPA1L, HSPA8, TUBA1B, TUBA1C); post-translational protein phosphorylation (AFP, C3, KNG1, SPARCL1); transcriptional regulation by small RNAs (H2AFX, H2AFZ, HIST1H2BK, RAN); and regulation of complement cascade (CPN1, C3, C7). Gene annotation for cellular components related to these signature genes by GeneCodis showed that C3, C7, FBLN1, GSN, H2AFX, H2AFZ, HSPA8, KNG1, LDHC, PGAM1, PRDX4, RAN, and TUBB belong to the extracellular exosome, whereas AFP, C3, C7, CPN1, FBLN1, GSN, HSPA8, KNG1, PGAM1, PRDX4, SPARC, SPARCL1, and TUBB were part of the extracellular region. Similarly, AFP, C3, CPN1, FBLN1, GSN, HIST1H2BK, HSPA8, KNG1, SPARC, and SPARCL1 belong to components of the extracellular space, and C3, GSN, HSPA8, PGAM1, and PRDX4 were found to be components of secretory granule lumen.

#### 3.2.5 Pathway analysis by Cytoscape

**Figure 5:**
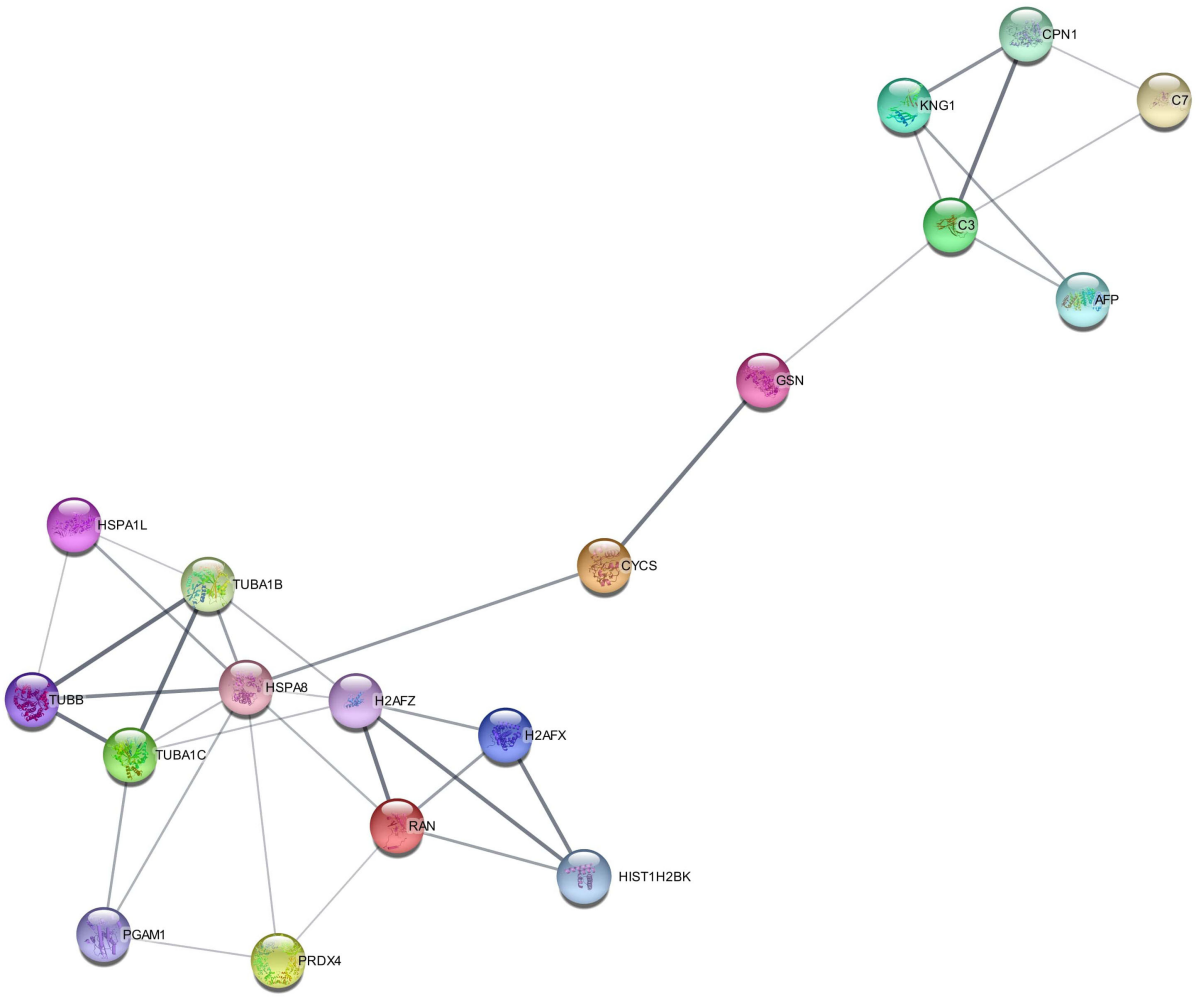
Protein–protein interaction (PPI) of signature differentially expressed proteins (DEPs) in the secretome of *Artemisia absinthium* whole-plant ethanolic extract-loaded NIPAAM-VP-AA (NVA-AA) nanoparticle-treated MCF-7 and MDA-MB-231 cell lines. This interactome was generated by the STRING module of Cytoscape software in which 18 out of 22 DEPs showed interaction with each other.

Protein–protein interaction of signature DEPs was generated by the STRING module of Cytoscape software as shown in **Figure 5**. Out of 22 signature DEPs, 18 showed interaction with each other. Interacting proteins belong to different biological processes including regulation of complement cascade (CPN1, C3, C7), cell cycle (H2AFX, H2AFZ, HIST1H2BK, RAN, TUBA1B, TUBA1C, TUBB), cellular responses to stimuli (CYCS, H2AFX, H2AFZ, HIST1H2BK, HSPA1L, HSPA8, TUBA1B, TUBA1C), innate immunity (C3, C7, CPN1, GSN, HSPA8, PGAM1, PRDX4, TUBB), and neutrophil degranulation (C3, GSN, HSPA8, PGAM1, PRDX4, TUBB).

#### 3.2.6 Functional annotation analysis by PANTHER

All the significant DEPs identified in NVA-treated MCF-7 and MDA-MB-231 were categorized based on their protein class, biological role, molecular functions, cellular component, and pathways involved. The results of this analysis are shown in **Supplementary Figure S4**. The majority of the proteins in MCF-7 and MDA-MB-231 are clustered as DNA binding, ATP-dependent activity, and functions corresponding to catalytic and regulatory activity. The biological classification of these proteins identified in MCF-7 and MDA-MB-231 revealed that these are majorly involved in cellular and metabolic processes as well as localization, signaling, biological adhesion, and immune system processes. In MCF-7 and MDA-MB-231, a large portion of proteins belong to the class of proteins that participate in chromatin binding or regulatory protein, while other proteins were cell adhesion molecules, chaperones, cytoskeleton proteins, transcriptional regulators, protein-binding activity modulators, and carrier proteins. A few additional proteins in MDA-MB-231 were categorized under the class of proteins responsible for RNA metabolism, extracellular matrix proteins, immunity system, and intercellular signal molecule. Pathway analysis in both cell lines shows that most of the proteins were involved in ATP synthesis, apoptosis signaling pathway, p53 pathway, blood coagulation, cadherin signaling, FAS signaling pathway, inflammation mediated by chemokine, cytokine, vitamin D metabolism, and Wnt signaling pathway. However, few proteins involved in EGF-receptor signaling, glycolysis, plasminogen activating cascade, and PI3 kinase pathway were additionally found in MDA-MB-231.

### 3.3 Real-time PCR

**Figure 6:**
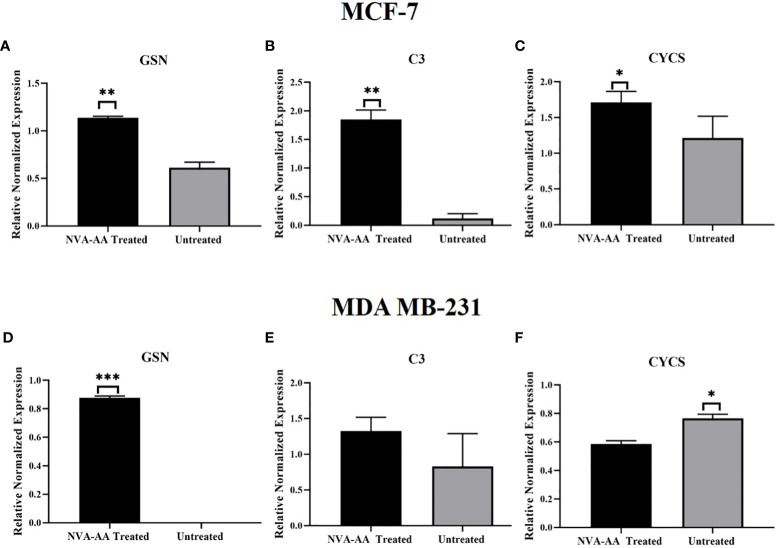
Quantitative real-time PCR analysis showing the relative expression of the mRNA transcripts of signature differentially expressed proteins (DEPs) belonging to cell cycle regulation **(A, D)** GSN, innate immune response **(B, E)** C3, and apoptosis **(C, F)** CYCS in MCF-7 and MDA-MB-231. The values are expressed as mean ± SEM (*P < 0.05, **P < 0.01, ***P < 0.001 when NVA-AA-treated cell lines were compared with untreated cell lines using unpaired *t*-test).

The gene expression analysis of GSN, C3, and CYCS in treated and untreated cell lines was conducted using quantitative real-time PCR (**Figure 6**). There was a significant upregulation of the GSN (*P* < 0.01, *P* < 0.001) in both NVA-AA NP-treated MCF-7 and MDA-MB-231 cell lines, indicating inhibition of cell cycle regulation; however, C3 (*P* < 0.01) showed a significant upregulation in NVA-AA NP-treated MCF-7 and a non-significant upregulation in NVA-AA NP-treated MDA-MB-231 cell lines, suggesting stimulation of innate immune response by NVA-AA NPs in MCF-7 and MDA-MB-231 cell lines. Moreover, CYCS was upregulated in treated MCF-7 and significantly downregulated (*P* < 0.05) in treated MDA-MB-231 suggesting induction of caspase-dependent and caspase-independent cell death by NVA-AA NPs.

## 4 Discussion

Temperature- and pH-responsive polymeric nanoparticles were previously utilized by our research group for site-specific drug delivery targeted against the tumor microenvironment comprised of low pH and high temperature accompanied by inflammation. We have reported the cytotoxic and anticancer potential of *A. absinthium* whole-plant ethanolic extract-loaded NIPAAM-VP-AA (NVA-AA) against the breast cancer cell lines MCF-7 and MDA-MB-231. NVA-AA NPs significantly inhibited cell proliferation and cell viability by inducing apoptosis in the treated cell lines (19). In the present study, we investigated differentially expressed secretory proteins in the media of NVA-AA NP-treated MCF-7 and MDA-MB-231 cell lines by employing nano LC-MS/MS to identify key pathways and easily accessible potential therapeutic protein targets affected by the cytotoxic action of NVA-AA NPs targeted against the tumor microenvironment. Our study revealed an altered secretome profile of *A. absinthium* whole-plant extract-loaded NVA-AA polymeric nanoparticle-treated MCF-7 and MDA-MB-231 cell lines. Several proteins involved in different biological functions and pathways were found to be significantly altered. Twenty-two signature DEPs were selected out of the total proteins which were most significantly altered due to the treatment. The selection of proteins was done by utilizing TCGA datasets available at GENT2 and GSCA.

Protein–protein interaction among 22 signature secretory proteins demonstrated that most affected signaling pathways accompanied by apoptosis induction and cell death by NVA-AA NPs in MCF-7 and MDA-MB-231 involved the regulation of complement cascade [carboxypeptidase N catalytic chain, complement components (C3 and C7)], modulation of the cell cycle [histone proteins (H2AFX, H2AFZ, HIST1H2BK), GTP-binding nuclear protein (RAN), tubulin (TUBA1B, TUBA1C, and TUBB)], cellular responses to stimuli [cytochrome c, histone (H2AFX, H2AFZ, HIST1H2BK), heat shock proteins (HSPA1L, HSPA8), tubulins (TUBA1B, TUBA1C)], triggering of innate immune response [complement components (C3, C7), CPN1, gelsolin, heat shock proteins (HSPA8), phosphoglycerate mutase 1, perioxidin-4, tubulin (TUBB)], and neutrophil degranulation [complement C3, gelsolin, heat shock proteins (HSPA8), phosphoglycerate mutase 1, perioxidin-4, tubulin (TUBB)].

Gelsolin is an actin-binding protein that causes conformational changes in actin monomers, facilitating filament polymerization. It also regulates the length of actin filament and other cellular functions including cell motility, division, and apoptosis by participating in different signaling pathways. It is a known tumor-suppressor gene that is downregulated in breast cancer due to epigenetic changes including histone acetylation during chromatin structural configuration (29). Previously, overexpression of gelsolin using recombinant adenovirus encoding wild-type gelsolin (Ad-GSN) arrested cell cycle at the G2/M phase, reduced cell division in bladder cancer cells (KU-7 and UMUC-2), and inhibited tumor growth in the orthotopic bladder cancer nude mouse model (30). Gelsolin reduced tumorigenicity in a human lung cancer cell line (PC10) *via* inhibiting phospholipases C (PLC)/protein kinase C (PKC) signal transduction pathway (31). Similarly, highly expressed gelsolin significantly inhibited invasion and metastasis in human colon carcinoma (CC) cells *in vitro* (32). Thus, these findings were similar to our results, i.e., upregulation of gelsolin due to NVA-AA treatment indicates that these NPs could cause cell death by impeding microtubule organization and cell division, irrespective of the breast cancer subtype. Similarly, cytochrome c somatic (CYCS) is an apoptotic biomarker whose expression was different in both treated cell lines, i.e., elevated in MCF-7 and reduced in MDA-MB-231. ROS-mediated programmed cell death is associated with the release of pro-apoptotic proteins like cytochrome c into the cytosol, which in turn, activates the caspase-dependent cascade of apoptosis (33). However, there are increasing studies demonstrating that different cytotoxic drugs are capable of inducing caspase-independent programmed cell death as a defense mechanism against external stimuli or stress (34). Apart from cytochrome c, apoptosis-inducing factor (AIF) is another protein with oxidoreductase activity and caspase-independent apoptogenic function, in which high ROS level triggers activation of PARP, translocation of AIF into the nucleus which in turn causes chromatin condensation, DNA fragmentation, and subsequent cell death (35). Increased level of CYCS in the extracellular space of treated MCF-7 in our study indicates the release of cytochrome c from the mitochondrial membrane associated with the induction of apoptosis by NVA-AA NPs. However, a reduced level of CYCS in treated MDA-MB-231 with significant cell death proposes the presence of cytotoxic drug-mediated caspase-independent cell death involving perturbation in ROS stability causing depolarization of mitochondrial membrane potential, the release of AIF, and finally cell death (36). Therefore, the anticancer effect of NVA-AA NPs causes differential apoptotic gene expression changes in MCF-7 and MDA-MB-231, due to differences in cell genotype.

Alpha-fetoprotein (AFP) is a glycoprotein that functions as a carrier protein for various ligands as well as the regulator of cellular growth of different cell types, which was overexpressed in NVA-AA NP-treated MCF-7 as well as MDA-MB-231 cell lines in our study. According to a study by Sierralta et al., the addition of an active site of AFP consisting of cyclized 9-amino acid peptide (cP) to MCF-7 breast cancer cells and estrogen-dependent ZR75-1 cells inhibited cell proliferation (37). Therefore, the upregulation of AFP along with cell proliferation inhibition induced by NVA-AA NPs proposes the possibility of these NPs interfering with the cell cycle regulation.

Immune cells are the key players whose pathological implications vary with the heterogeneous pattern of gene expression in cancer initiation, progression, and response to cytotoxic drugs. Complement components are one of the crucial factors in cancer that specifically binds to cells at immune surveillance facilitating the removal of apoptotic cells by macrophages (38). On the contrary, complement components can also contribute to tumor growth by supporting angiogenesis and causing chronic inflammation (39). Among several components, a high level of C3 has been considered a diagnostic marker in breast and lung cancer (40). Previously, upregulation of C3 due to docetaxel/epirubicin-based chemotherapy suggested it as a good predictive marker of therapeutic response in breast cancer (41). Thus, complement pathway-mediated regulation of tumor growth varies among different tumor types. Monteran et al. reported increased expression of C3 and C7 in lung fibroblasts of doxorubicin-treated mice and proposed their involvement in the formation of the immunosuppressive tumor microenvironment (42). In the present study, increased levels of two isoforms of complement components, i.e., complement component C3 (in MCF-7 and MDA-MB-231) and C7 (MDA-MB-231) by NVA-AA NPs, were seen in both cell lines indicating activation of the complement pathway *via* triggering the innate immune system. Thus, our data suggest that cytotoxicity caused by NVA-AA NPs elicited activation of complement cascade due to induced apoptosis.

Tubulins are cytoskeletal proteins whose alpha and beta subunits heterodimerize for the microtubule assembly required for cell division. Agents affecting tubulin dynamics have been utilized as an ideal approach for chemotherapeutics (43, 44). NVA-AA NPs resulted in reduced expression of signature DEPs from the tubulin family including TUBB, TUBA1B, and TUBA1C, confirming its ability to hinder the formation of microtubules by binding to tubulins, eventually causing cell death through apoptosis. Heat shock protein HSP 90-beta (HSP90AB1) was the most significantly downregulated secretory protein in MDA-MB-231 after the NVA-AA NP treatment. Therefore, our results were consistent with the study by Rasouliha et al. that showed the downregulation of tubulin genes and HSP90AB1 due to doxorubicin treatment in MCF-7 cells (45). Therefore, this decrease in tubulin levels would have reduced the rate of cell division in MDA-MB-231. On the contrary, a few tubulin genes (TUBB, TUBA1B) were overexpressed in NVA-AA NP-treated MCF-7 cell lines. Cytoskeletal-interacting agents can either stabilize or destabilize the microtubule organization, i.e., enhanced tubulin expression represents an increase in tubulin monomer required for polymerization due to its depletion, whereas reduced tubulin expression demonstrates the increase in the tubulin monomer due to inhibition of polymerization (46). Thus, we anticipate that these differences between both cell lines could be due to the difference in the nature of interaction of these NPs with different breast cancer subtypes.

Histone variants including H2AFZ and H2AFX were downregulated in MDA-MB-231, whereas HIST1H2BK was downregulated in both MCF-7 and MDA-MB-231 cell lines after NVA-AA NP treatment. Histones are critical drivers of DNA packaging into the nucleosome which is differentially regulated throughout the cell cycle. H2AFZ is an oncogene that was overexpressed in breast cancer patients with adverse clinical outcomes (47). Similarly, the H2AFX variant was also found to be associated with the initiation and progression of breast cancer (48). Previously, upregulation of HIST1H2BK in TNBC MDA-MB-231 was found to be correlated with chemoresistance induced by doxorubicin (49). HIST1H2BK was one of the most significantly downregulated differentially expressed histone proteins due to Canady Helios Cold Plasma™ (CHCP) treatment of breast cancer cells where CHCP mainly degrades histone proteins during the early S phase of the cell cycle (50). Therefore, apparent cell death associated with histone regulation by NVA-AA NPs in our study determines its ability to cause chromatin instability and aberrant cell cycle regulation, subsequently affecting cell survival.

Heat shock proteins (HSPs) are stress-responsive molecules that are required for the stabilization of oncogenes, their transcriptional regulation, protein folding, and cell cycle maintenance (51). Two signature HSPs were altered by NVA-AA NPs in MDA-MB-231, where the expression of heat shock cognate 71 kDa protein (HSPA8) was reduced and heat shock 70 kDa protein 1-like (HSPA1L) was elevated. Sporadic breast cancer is found in association with a deletion mutation in HSPA8, located on chromosome 11q23.3 (52). Overexpressed HSPA8 was also considered a poor prognosis marker of breast cancer (53). HSPA1L was one of the downregulated genes after gemcitabine treatment used against breast cancer (54). In our study, NVA-AA NPs stimulated altered expression of these HSPs as a consequence of modifications in gene transcriptional regulation.

Phosphoglycerate mutase 1 (PGAM1) is a key metabolic enzyme in the glycolysis pathway whose overexpression is associated with breast cancer. However, Zhang et al. demonstrated the non-metabolic function of PGAM1, which is involved in modulating actin filament organization, cell motility, migration, and enhancing cancer progression (55). Previously, methotrexate (MTX), a chemotherapeutic drug, has shown downregulation of PGAM1 in the MCF-7 cell line (56). In the present study, PGAM1 was also significantly downregulated, which can be corroborated by the reduction in cell proliferation and viability in MDA-MB-231 caused by NVA-AA NPs.

GTP-binding nuclear protein RAN is a regulatory protein mainly involved in the exchange of molecules across the nucleus membrane by undergoing GTP/GDP-bound conformation changes. It also plays an important role during the cycle by controlling the assembly of the mitotic spindle, cell cycle checkpoint, and nuclear envelope formation. Upregulation of RAN has been found in many tumor types, including breast tumors, contributing to cancer invasion and poor prognosis (57). An *in-vitro* investigation by Sheng et al. showed inhibition of cell proliferation, motility, and cell cycle arrest in MDA-MB-231 cell lines by treatment with si-RNA directed against RAN (RAN-si-RNA) (58). Therefore, our findings with significant cell death accompanied by downregulation of RAN in NVA-AA NP-treated MDA-MB-231 implicated the potential of RAN to be considered as a therapeutic target.

Kininogen 1 (KNG1) is a crucial component of the blood coagulation system whose expression is lowered in a variety of cancers including breast, ovarian, and prostate. It is known as a metastasis inhibitor, capable of inhibiting angiogenesis, invasion, and migration of human prostate cancer (59). Downregulated KNG1 was also identified in metastatic tumor-draining lymph from metastatic mammary carcinoma through LC/MS proteomic analysis (60). To our knowledge, this is the first study reporting the upregulation of KNG1 in MDA-MB-231 after NVA-AA NP treatment, representing it as a promising therapeutic target for inhibiting cancer invasion and metastasis. Carboxypeptidase N catalytic chain (CPN1) exhibits peptidase activity by cleaving the substrate peptide, KNG1, at the carboxy-terminal arginine residue. A low level of CPN1 was observed in MDA-MB-231 and MCF-7 than in non-tumorigenic MCF-10A cell lines (61). CPN1 was considered a tumor biomarker for assessing invasion and metastasis status in breast cancer patients (62). Similarly, protein–protein interaction in our study also demonstrated a direct interaction between upregulated KNG1 and CPN1 supporting the existing data.

Fibulin-1 (FBLN1) is an ECM-associated glycoprotein, localized in the basement membrane and interacting with other components like laminin. FBLN1 has a critical role in epithelial-to-mesenchymal transition (EMT) during cancer due to its capability of regulating cell differentiation, adhesion, migration, and proliferation. High as well as aberrant expression of FBLN1 was assessed in breast carcinoma (63). However, the elevated expression level of FBLN1 in fibrosarcoma-derived cells has demonstrated inhibition of tumor formation in nude mice as well as invasion in the gels of reconstituted basement membrane extracts (64). Fibulin-1 has previously shown the ability to inhibit cell migration, invasion, and motility in melanoma, epidermoid carcinoma, and breast carcinoma cell lines through inhibition of ERK activation based on fibronectin-specific mechanisms (65). Silencing of fibulin-1 has shown an increment in cell proliferation in MCF-7 *in vitro*, and a low proliferation index was found in fibulin-1-expressing breast cancer tissue samples (66). Thus, the role of fibulin-1 in cancer progression may vary with cancer type. Therefore, our study corroborates with these studies as the overexpression of fibulin-1 in the MDA-MB-231 cell line was associated with the inhibition of cell proliferation and migration by destabilizing ECM structure and integrity.

Cellular damage caused by oxidative stress and redox status imbalance is closely associated with cancer cell proliferation and survival. Peroxiredoxins 4 (PRDX4) is one of the antioxidant enzymes specifically located in the endoplasmic reticulum (ER) and secreted in the extracellular space. It has been suggested that PRDX4 has a potential role in tumor initiation, progression, chemoresistance against docetaxel, and disease recurrence. High expression of PRDX4 has been reported in a variety of cancers including breast cancer; on the other hand, increased tissue level of PRDX4 was found to be correlated with a better survival rate in breast cancer patients (67). However, our study reporting downregulation of the antioxidant enzyme PRDX4 after treatment is in agreement with the OS analysis provided by TCGA datasets in GETN2 representing the association of low expression with better survival, thus helping us to postulate that oxidative stress burden was reduced due to the antioxidant potential of *A. absinthium* extract encapsulated in NVA NPs.

Secreted protein acidic and rich in cysteine (SPARC) binds with ECM components and growth factors mediating cell–ECM interaction, modulating different cellular processes, cell microenvironment, adhesion, and drug resistance (68). Contradictory regulation of SPARC has been documented in the initiation and progression of cancer, although the majority of existing literature reveals that downregulation of SPARC is closely associated with the aggressive phenotype of breast cancer progression (69, 70). In a study by Nagai et al., negative SPARC immunostaining of tumors was associated with poor prognosis in luminal A and triple-negative breast cancer patients (71). Previously, SPARC has been proposed as a tumor-suppressor protein that can induce apoptosis and inhibit angiogenesis by reducing VEGF expression (72, 73). Therefore, overexpression of SPARC by NVA-AA NPs supports the previous findings reporting the induction of apoptosis associated with high expression of SPARC in breast cancer. Similarly, secreted protein acidic and rich in cysteine-like protein 1 (SPARCL1) was another ECM-glycoprotein whose reduced expression was reported in human breast cancer tissues, and NVA-AA NP treatment caused an increase in the SPARCL1 expression (74). SPARCL1 is 62% identical to SPARC and contains conservative structural domains with functional similarity (75). It has also been considered a tumor suppressor as well as an oncogene (76, 77); thus, altered regulation of SPARCL1 after treatment can be useful in implicating it as a therapeutic target in breast cancer.

Lactate dehydrogenase C (LDHC) is an isoform of the lactate dehydrogenase (LDH) family whose elevated expression was observed in serum-derived exosomes as well as tissue of breast cancer patients and associated with adverse clinical outcomes, poor survival, and remission (78). It is also considered a tumor-associated antigen due to its immunogenic nature promoting cytotoxic immune response in breast cancer (79). In a study by Naik et al., LDHC silencing led to the abrupt progression of the cell cycle with substantial expression alteration in cell cycle checkpoint components, disassembly of nuclear and microtubule components, and DNA damage regulators in breast cancer (80). Downregulation of LDHC in our study along with cell cycle arrest at the G0/G1 phase corroborates with the observations of the previous study by Naik et al., further confirming LDHC to be used as a potential therapeutic target modulating DNA integrity.

Angiogenesis in cancer progression is highly regulated through interactions of ECM constituting the tumor microenvironment. In the present study, THBS1 was highly expressed in NVA-AA-treated MCF-7 cell lines, which is known as a potential inhibitor of angiogenesis (81). THBS1 is a glycoprotein that can strongly bind to ECM, affecting their structure and function *via* modulating direct as well as indirect interactions between other secretory factors (82, 83). THBS1 could inhibit angiogenesis by modifying the assembly of the actin cytoskeleton and focal adhesion of endothelial cells which would lead to the inhibition of cellular migration and invasion (84). A few genes belonging to the family of keratins (KRT1, KRT10, KRT2, KRT77) were downregulated in our study, which are filament proteins required for the maintenance of epithelial cells and have a role in cell motility, protein synthesis and regulation of signaling pathways.

Evaluation of the correlation between the mRNA expressions of 22 signature DEPs in MCF-7 and MDA-MB-231 cell lines versus the sensitivity of cancer cells toward small-molecule drugs of GDSC and CTRP was done. The dysregulated proteins in NVA-AA-treated MCF-7 and MDA-MB-231 cells, viz., KNG1, TUBA1C, FBLN1, AFP, PRDX4, SPARC, GSN, and C3, were found to have a positive correlation of their mRNA expressions with the GDSC drug THZ-2-102-1. Similarly, KNG1 and AFP with YM156, AFP with ZG-10, RAN with WH-4-023, and RAN and HSPA8 with TGX221 were found to be positively correlated. Since downregulation of TUBA1C and PRDX4 proteins was observed only in NVA-AA-treated MDA-MB-231 cells, their downregulated expressions might make MDA-MB-231 sensitive to NVA-AA treatment in the same fashion as shown by cancer cells to THZ-2-102-1 treatments. However, overexpression of GSN, C3, and AFP due to NVA-AA NPs in both cell lines (MCF-7 and MDA-MB-231) displayed negative correlations with WH-4-023, TGX221, and pazopanib. Thus, their overexpression might increase the sensitivity to NVA-AA treatment similar to WH-4-023, TGX211, and pazapanib treatments to other cancer cells. Moreover, the change in the expression of TUBA1C, PRDX4, LDHC, C7, and KNG1 due to NVA-AA treatment corroborated with the sensitivity provided by CTRP drugs to cancer cells (85). Thus, differential expression of proteins by NVA-AA NPs imparting sensitivity to the two cancer cells could be correlated to their sensitivities with the GDSC and CTRP drugs in different cancer cells.

Immune cell infiltration (Tfh, NK, CD4_T, CD8_T, NKT, B cell) showed positive correlations with the expressions of the dysregulated signature genes/proteins in NVA-AA-treated MCF-7 and MDA-MB-231 cell lines. These immune cells were reported to be involved in antitumor immune responses and also considered important targets in immunotherapy (86–90). However, immune cells, viz., neutrophil, Th17, nTreg, and macrophage, showed negative correlations of their infiltrations with the expressions of the dysregulated signature genes/proteins in both NVA-AA-treated cell lines. These immune cells were mainly found to be involved in angiogenesis, cancer promotion, metastasis, poor prognosis, and relapse of tumor cells, which are the hallmarks of cancer manifestations and progression (91–95).

The limitations of our *in-vitro* study include further experimental validation at the *in-vivo* level due to variation in the tumor microenvironment, the presence of certain biological barriers at the cell line and organism level, and bioinformatics analysis findings, which may further be comprehended through *in-vitro* and *in-vivo* experiments. Remarkably, validating and establishing *A. absinthium* extract-loaded NVA-AA NPs as the drug carrier ensuring site-specific drug delivery would increase the therapeutic effectiveness and lessen side effects related to the existing anticancer payload associated with currently available treatments. However, the clinical application of NVA-AA NPs against breast cancer requires more vigorous preclinical studies to ensure its efficacy and safety at an individual level. Also, it will be interesting to investigate the effect of these NPs on other cancer types as well.

This study has a fundamentally exploratory aim which was focused on the secretome profiling to unravel the altered processes and pathways in MCF-7 and MDA-MB-231 breast cancer cell lines caused due to the cytotoxicity of *A. absinthium* whole-plant extract-loaded NVA-AA polymeric nanoparticles. We can conclude that cytotoxicity by NVA-AA NPs was mainly associated with their capacity to interfere with the cytoskeletal dynamics, cell cycle regulators, cell–cell interaction, intracellular trafficking, cell polarization, and migration of cancer cells, irrespective of the breast cancer subtype.

## Data availability statement

The datasets presented in this study can be found in online repository named “PRIDE database”, with accession number “PXD042365”.

## Ethics statement

Ethical approval was not required for the studies on humans in accordance with the local legislation and institutional requirements because only commercially available established cell lines were used. Ethical approval was not required for the studies on animals in accordance with the local legislation and institutional requirements because only commercially available established cell lines were used.

## Author contributions

SK did the investigation, data curation, formal analysis, methodology, visualization, validation, writing—original draft, and writing—review and editing. MM was involved in data curation, methodology, validation, and writing—review and editing. IM did the investigation, visualization, and writing—review and editing. SS was involved in the methodology and writing—review and editing. SW did the conceptualization, methodology, supervision, project administration, funding acquisition, validation, visualization, and writing—review and editing. All authors contributed to the article and approved the submitted version.
