# Supplementary material for: Secretome profiling of Artemisia absinthium extract-loaded polymeric nanoparticle-treated MCF-7 and MDA-MB-231 revealed perturbation in microtubule assembly and cell migration
Source: Front Oncol. 2023 Aug 31;13:1209168. doi: 10.3389/fonc.2023.1209168 (PMC10502211; doi:10.3389/fonc.2023.1209168)
Supplement: Supplementary file 1 [file DataSheet_1.docx]

**Supplementary file**

**Table S1:** List of primers used for relative expression analysis by real time PCR

| **Gene** | **Template** | **Forward primer** | **Reverse primer** | **Tm** | **Product size (bp)** | **References** |
| --- | --- | --- | --- | --- | --- | --- |
| *GSN* | cDNA | GGTGTGGCATCAGGATTCAAG | TTTCATACCGATTGCTGTTGGA | 58.78 | 199 | Mayanagi et al. 2008 [2] |
| *CYCS* | cDNA | CAAAAACAAGGGCCAGATGT | GCTACCACACTGGACAGCAA | 58.62 | 136 | Kim et al. 2016 [3] |
| *C3* | cDNA | GGAGCAGTCAAGGTCTACGC | GCTTTCCATCCTCCTTTTCC | 58.41 | 82 | Kobayashi et al. 2012 [4] |

**Table S2:** Fold change (LOG2FC) and p-value of signature DEP selected from NVA-AA NPs treated MCF-7 and MDA MB-231 cell lines with significant expression change as compared with their expression in Luminal A and TNBC subtype provided by GENT2.

| **S.No.** | **Gene symbol** | **Fold change (LOG2FC) across Luminal A and TNBC subtypes** | **P value** |
| --- | --- | --- | --- |
| 1 | Tubulin beta chain (TUBB) | 0.23 | <0.001 |
| 2 | Cytochrome C somatic (CYCS) | 1.169 | <0.001 |
| 3 | Histone H2A.Z (H2AFZ) | 1.014 | <0.001 |
| 4 | Tubulin alpha-1C chain (TUBA1C) | 0.497 | <0.001 |
| 5 | Tubulin alpha-1B chain (TUBA1B) | 0.383 | <0.001 |
| 6 | Gelsolin (GSN) | -0.386 | <0.001 |
| 7 | Alpha-fetoprotein (AFP) | -0.37 | <0.001 |
| 8 | SPARC | -0.81 | <0.001 |
| 9 | Fibulin-1 (FBLN1) | -0.912 | <0.001 |
| 10 | Phosphoglycerate mutase 1 (PGAM1) | 0.769 | <0.001 |
| 11 | Complement component C7 (C7) | -1.435 | <0.001 |
| 12 | SPARC-like protein 1 (SPARCL1) | -0.931 | <0.001 |
| 13 | Carboxypeptidase N catalytic chain (CPN1) | -0.38 | <0.001 |
| 14 | Peroxiredoxins 4 (PRDX4) | 1.704 | <0.001 |
| 15 | Lactate dehydrogenase C (LDHC) | 1.217 | <0.001 |
| 16 | GTP-binding nuclear protein Ran (RAN) | 0.693 | <0.001 |
| 17 | Kininogen 1 (KNG1) | -0.147 | 0.029 |


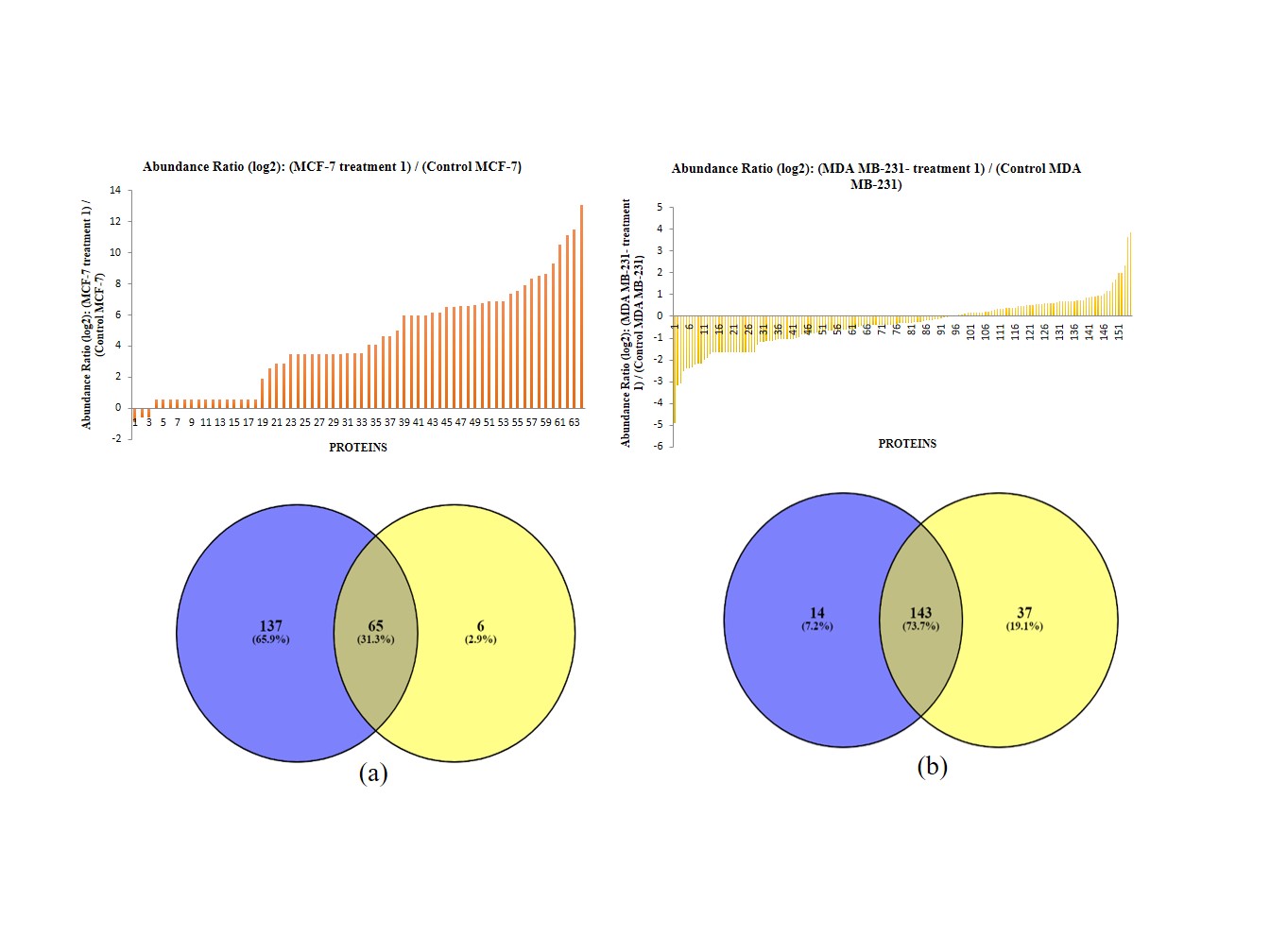


**Figure S1:** Graphical representation and Venn diagram showing significantly differentially expressed proteins identified in MCF-7 [Figure 1 (a)] and MDA MB-231 cell lines [Figure 1 (b)], respectively.


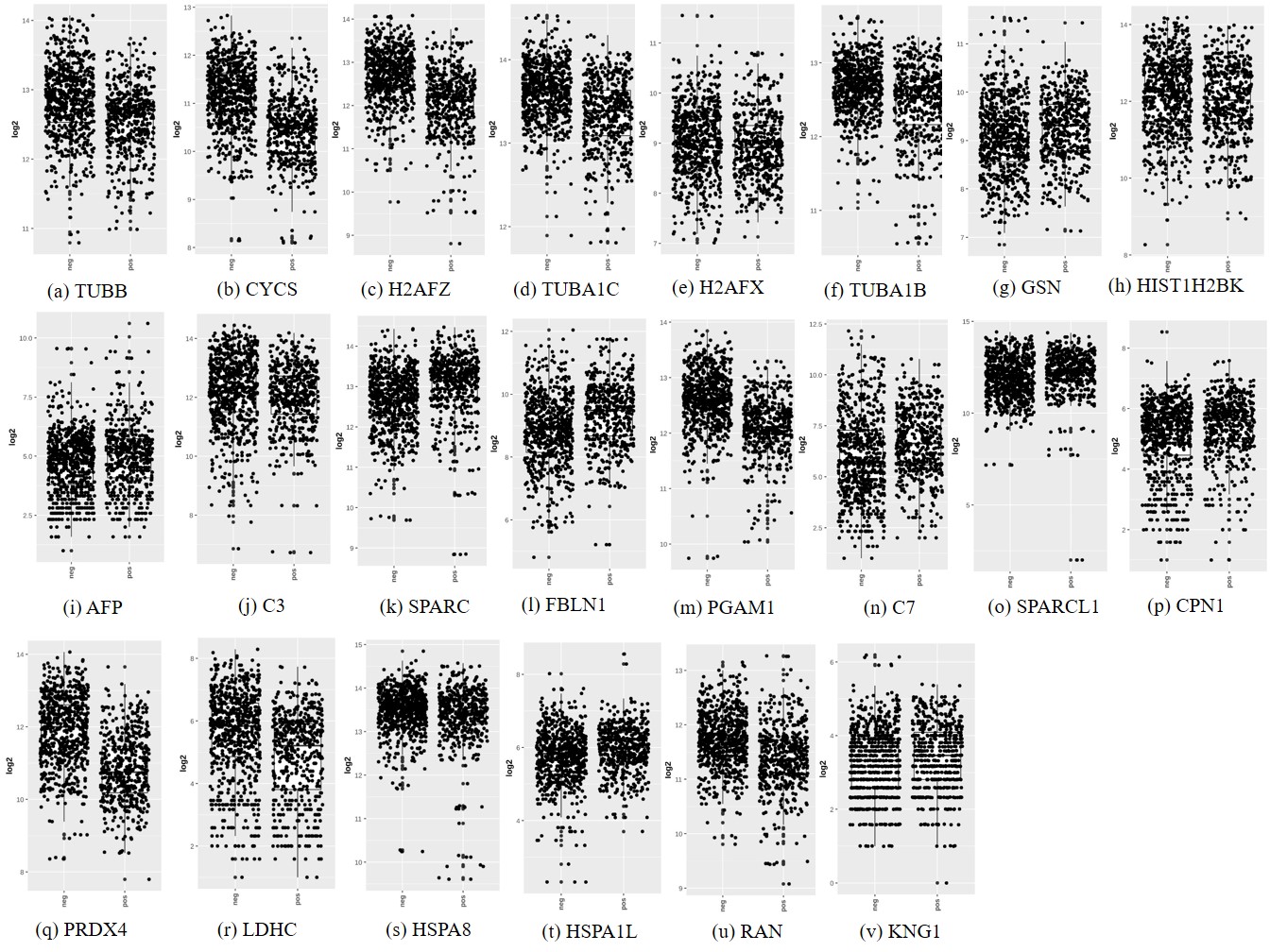


**Figure S2:** Expression profile of 22 signature DEP in ER positive and negative Breast Cancer patients retrieved from GENT2: These signature DEPs were selected from NVA-AA NPs treated MCF-7 (ER-positive) and MDA MB-231 (ER-negative) cell lines due to significant reversal of their expression as compared with expression profile in ER positive and negative Breast Cancer patients obtained from GENT2.


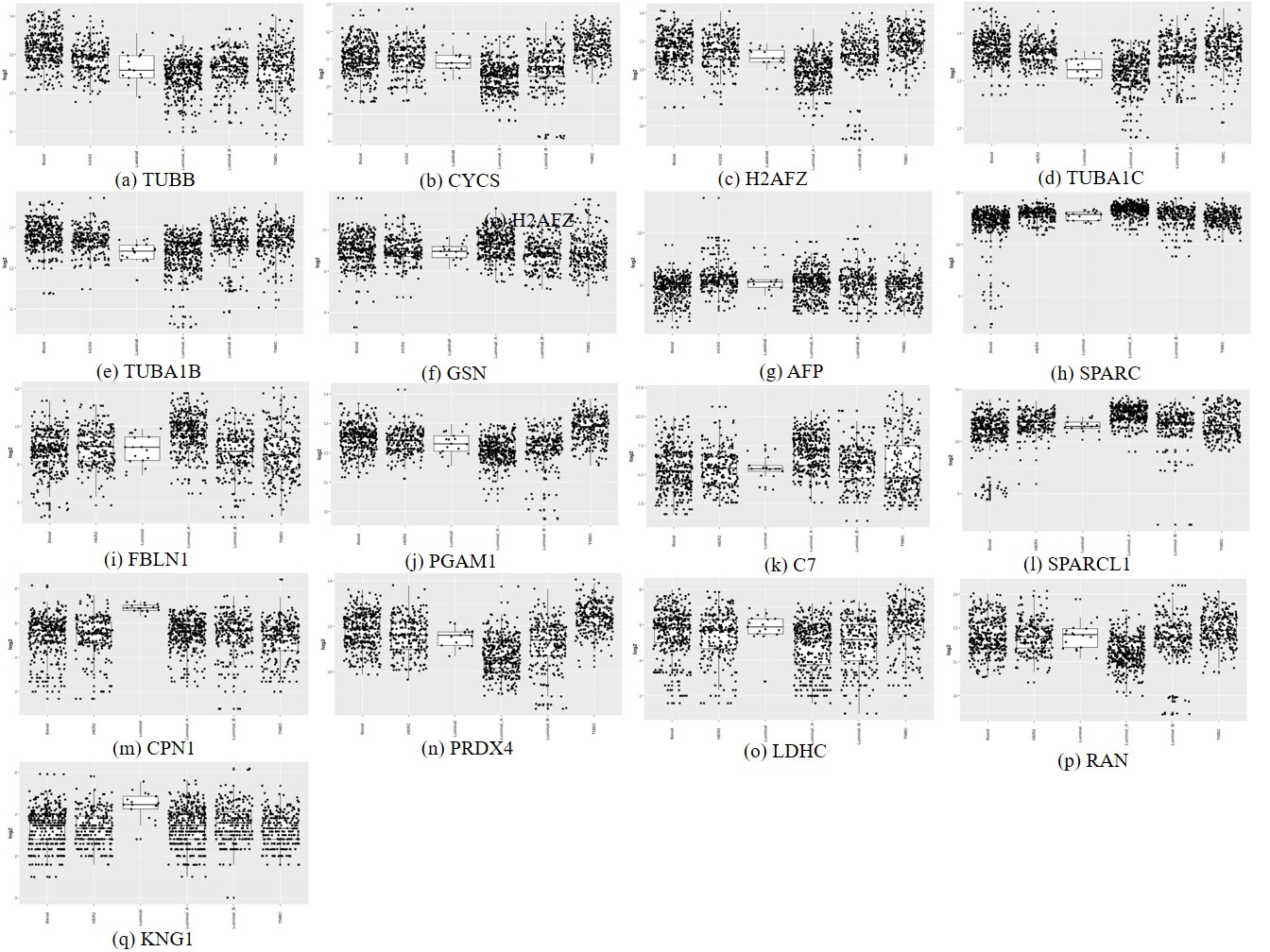


**Figure S3:** Expression profile of suitable signature DEPs from MCF-7 and MDA MB-231 whose expression after NVA-AA NPs treatment were reversed as compared across Luminal A and TNBC subtype. High expression of TUBB, CYCS, H2AFZ, TUBA1C, TUBA1B, PGAM1, PRDX4, LDHC, and RAN and low expression of SPARC, FBLN1, C7, SPARCL1, CPN1, and KNG1 in TNBC subtype (obtained from GENT2) was down-regulated and up-regulated in NVA AA NPs treated MDA MB-231, respectively. However, low expression of GSN and AFP was up-regulated in both, treated MCF-7 and MDA MB-231. These findings supports the therapeutic efficiency of these NPs.


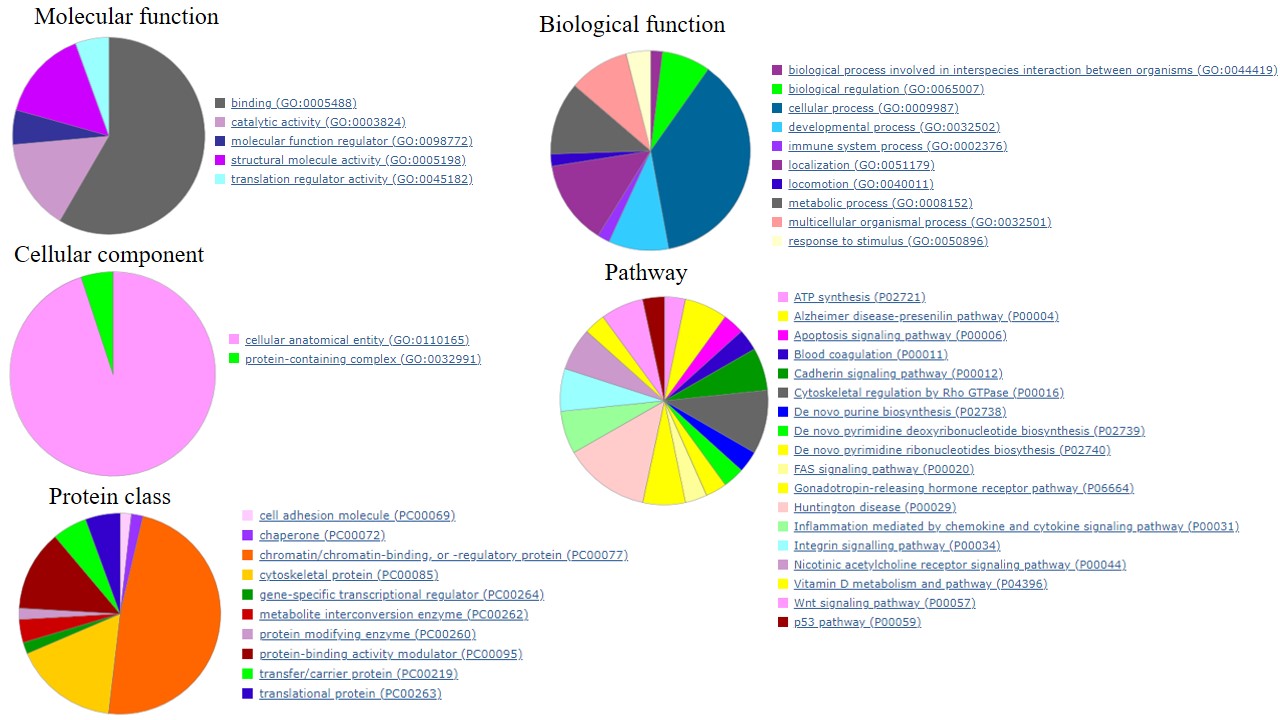


**(a)**

**
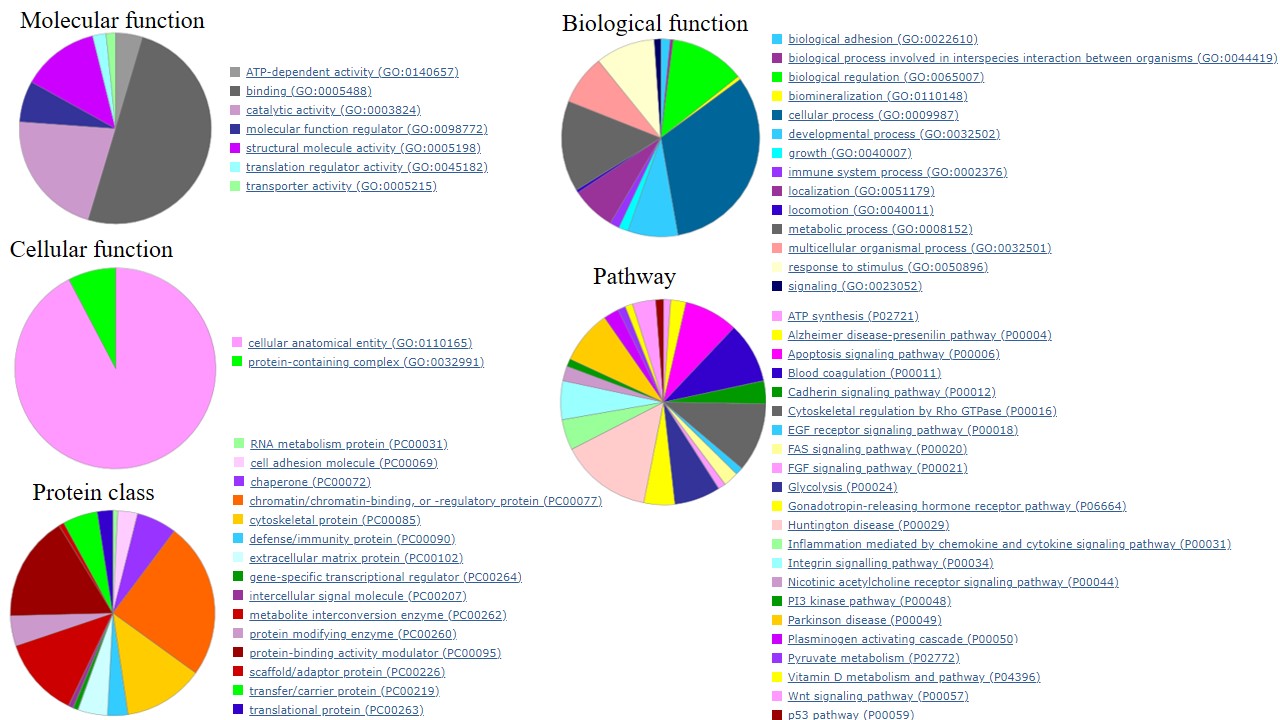
**

**(b)**

**Figure S4:** Gene ontology of significant differentially expressed protein (DEP) identified in NVA-treated (a) MCF-7 and (b) MDA MB-231 were categorized based on their protein class, biological role, molecular functions, cellular component, and pathways involved by PANTHER software.

**References**

1. Mayanagi T, Morita T, Hayashi KI, Fukumoto K, Sobue K. Glucocorticoid receptor-mediated expression of caldesmon regulates cell migration via the reorganization of the actin cytoskeleton. Journal of Biological Chemistry. 2008 Nov 7;283(45):31183-96.
2. Kim SH, Ho JN, Jin H, Lee SC, Lee SE, Hong SK, Lee JW, Lee ES, Byun SS. Upregulated expression of BCL2, MCM7, and CCNE1 indicate cisplatin-resistance in the set of two human bladder cancer cell lines: T24 cisplatin sensitive and T24R2 cisplatin resistant bladder cancer cell lines. Investigative and clinical urology. 2016 Jan 1;57(1):63-72.
3. Kobayashi H, Yamashita Y, Iwase A, Yoshikawa Y, Yasui H, Kawai Y, Uchida K, Uno N, Akatsuka S, Takahashi T, Kikkawa F. The ferroimmunomodulatory role of ectopic endometriotic stromal cells in ovarian endometriosis. Fertility and sterility. 2012 Aug 1;98(2):415-22.
